# A cross-model single-cell atlas reveals conserved involvement of osteopontin in polycystic kidney disease

**DOI:** 10.1172/jci.insight.207157

**Published:** 2026-07-28

**Authors:** Sarah J. Miller, Hua Zhong, Weidong Wu, Audrey M. Cordova, Morgan E. Yashchenko, Alex Yashchenko, Zhang Li, Daniyal J. Jafree, Chelsea N. Zimmerman, Christa I. DeVette, Vicki Do, Maya E. Hignite, Yohan Park, Fariha Nusrat, Bibi Maryam, Sizhao Lu, Xiaoyan Li, Jenny R. Gipson, Xiaogang Li, David A. Long, Mary C.M. Weiser-Evans, Bradley K. Yoder, Benjamin D. Cowley, Katharina Hopp, Jason R. Stubbs, Qin Ma, Anjun Ma, Kurt A. Zimmerman

**Affiliations:** 1 Department of Internal Medicine, Division of Nephrology, University of Oklahoma Health Sciences Center, Oklahoma City, Oklahoma, USA.; 2 Department of Biomedical Informatics, College of Medicine, and; 3 Pelotonia Institute for Immuno-Oncology, The James Comprehensive Cancer Center, The Ohio State University, Columbus, Ohio, USA.; 4 Department of Cell, Developmental, and Integrative Biology, University of Alabama at Birmingham, Birmingham, Alabama, USA.; 5 Developmental Biology & Cancer Research & Teaching Department, UCL Great Ormond Street Institute of Child Health, and; 6 UCL Centre for Kidney and Bladder Health, University College London, London, United Kingdom.; 7 Cellular Genetics Programme, Wellcome Trust Sanger Institute, Hinxton, United Kingdom.; 8 Department of Pediatrics, University of Oklahoma Health Science Center, Oklahoma City, Oklahoma, USA.; 9 Division of Nephrology, Boston Children’s Hospital, Boston, Massachusetts, USA.; 10 Department of Medicine, Division of Renal Diseases and Hypertension, University of Colorado Anschutz Medical Campus, Aurora, Colorado, USA.; 11 Department of Internal Medicine, and; 12 Department of Biochemistry and Molecular Biology, Mayo Clinic, Rochester, Minnesota, USA.; 13 College of Medicine Core Facilities, University of Oklahoma Health Sciences Center, Oklahoma City, Oklahoma, USA.; 14 The Jared Grantham Kidney Institute, and; 15 Division of Nephrology and Hypertension, Department of Internal Medicine, University of Kansas Medical Center, Kansas City, Kansas, USA.

**Keywords:** Cell biology, Immunology, Nephrology, Biomarkers

## Abstract

Polycystic kidney disease (PKD) arises from mutations in cilia-associated genes, such as *PKD1* and *PKD2*, expressed in renal epithelial cells, leading to progressive kidney dysfunction and end-stage kidney disease. Patients with PKD exhibit significant heterogeneity in disease progression, largely due to genetic and environmental modifiers. Like patients, mouse models of PKD also exhibit significant heterogeneity with regards to the gene mutated, age of disease onset, and rate of disease progression. To elucidate the cellular and molecular consequences of these variables, we constructed an integrated single-cell RNA sequencing (scRNA-seq) atlas across mouse models of PKD, mapping changes in cell type composition, gene expression, and intercellular signaling networks across the whole atlas and within individual models. Across models, scRNA-seq data revealed increased *Spp1* (osteopontin) expression and signaling from PKD-enriched clusters. Global deletion of *Spp1* in *Pkd1*^RC/RC^ mice resulted in a modest reduction in cyst severity and improved kidney function. From these studies, we created a freely available, searchable website (https://bmblx.bmi.osumc.edu/scPKD/) that can be used to identify cross- and intra-model changes in gene expression, guiding researchers to new therapeutic targets for treating PKD.

## Abstract 2

In this study we have created a cross-model single-cell atlas of polycystic kidney disease (PKD) through which we identified the conserved involvement of osteopontin (Spp1) across models as well as human patients.

## Graphical abstract

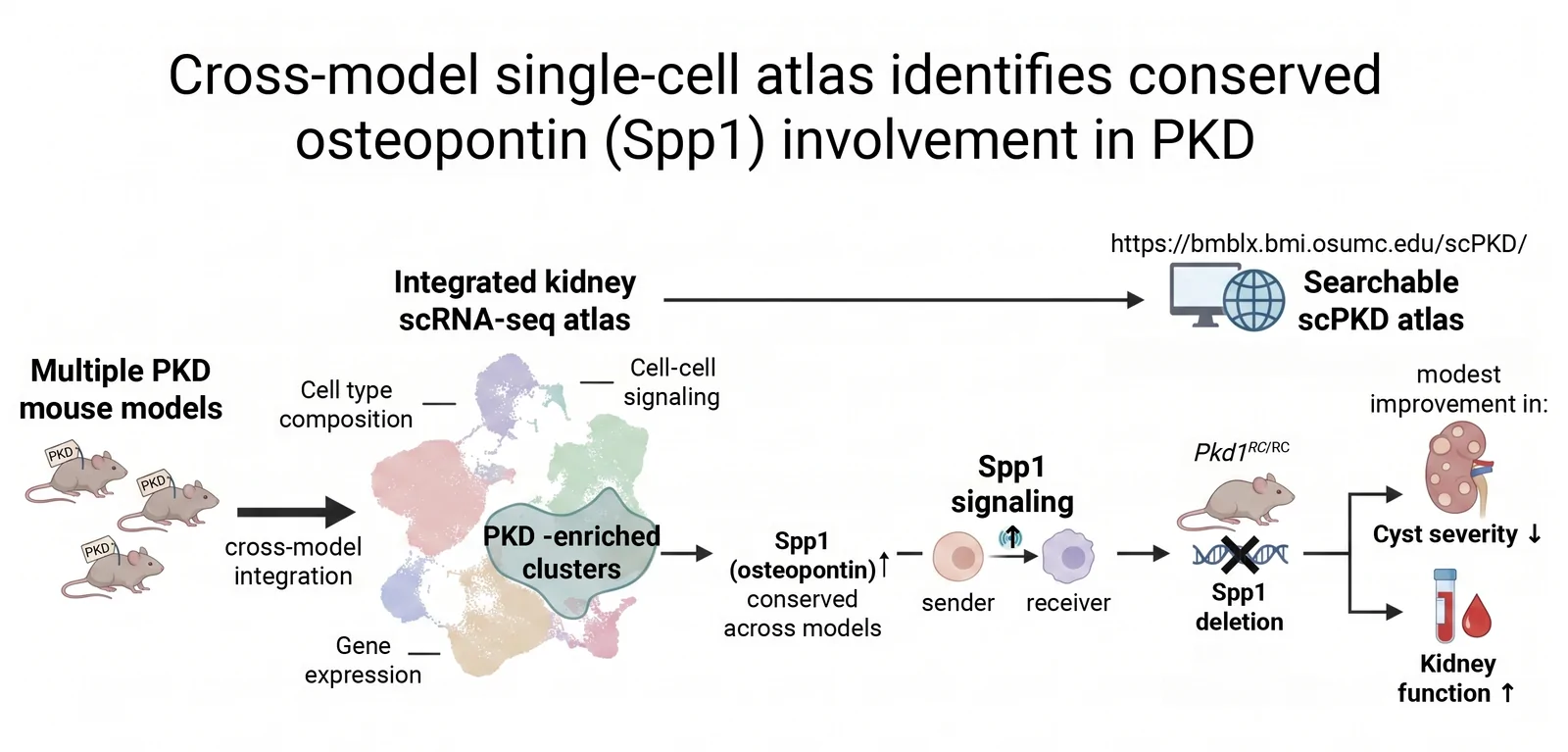

## Introduction

Autosomal dominant polycystic kidney disease (ADPKD) is caused by mutations in cilia-related genes, including *PKD1* and *PKD2*, and results in the development and growth of fluid-filled cysts throughout the kidney, eventually leading to end-stage kidney disease (1–3). Patients with ADPKD have significant heterogeneity in the rate of disease progression, largely owing to the spectrum of genetic variants and environmental modifiers (4). Like patients, mouse models of PKD also exhibit significant heterogeneity with regards to the gene mutated, age of disease onset, and rate of disease progression. Other differences between models include promoters that drive Cre recombinase activity, which can range from nephron-specific (*Pax8*, *Ksp*, or *Pkhd1*) to global (*Cagg*). Further adding to the complexity is the epistatic relationship between PKD genes (*Pkd1* and *Pkd2*) and primary cilia, as mice lacking both primary cilia and PKD-associated genes have less severe disease compared with mice lacking only PKD-associated genes (5). Collectively, the variation in PKD models significantly impairs our understanding of shared and model-specific cellular and molecular features of PKD.

In this study, we generated a cross-model, single-cell RNA sequencing (scRNA-seq) atlas to understand how PKD-associated mutations impact the frequencies of kidney cell types and their molecular profiles across and within individual PKD models. Using our integrated atlas, we identified cross-model dysregulation of osteopontin (SPP1) signaling and validate the functional importance of this observation by showing that genetic deletion of *Spp1* modestly improves disease severity in the orthologous *Pkd1*^RC/RC^ mouse model. We also created a freely available, searchable website (https://bmblx.bmi.osumc.edu/scPKD/) that can be used to identify cross- and intra-model specific changes in gene expression, guiding researchers to new therapeutic targets for treating PKD.

## Results

### Generation of a cross-model, scRNA-seq atlas of mouse PKD.

**Figure 1:**
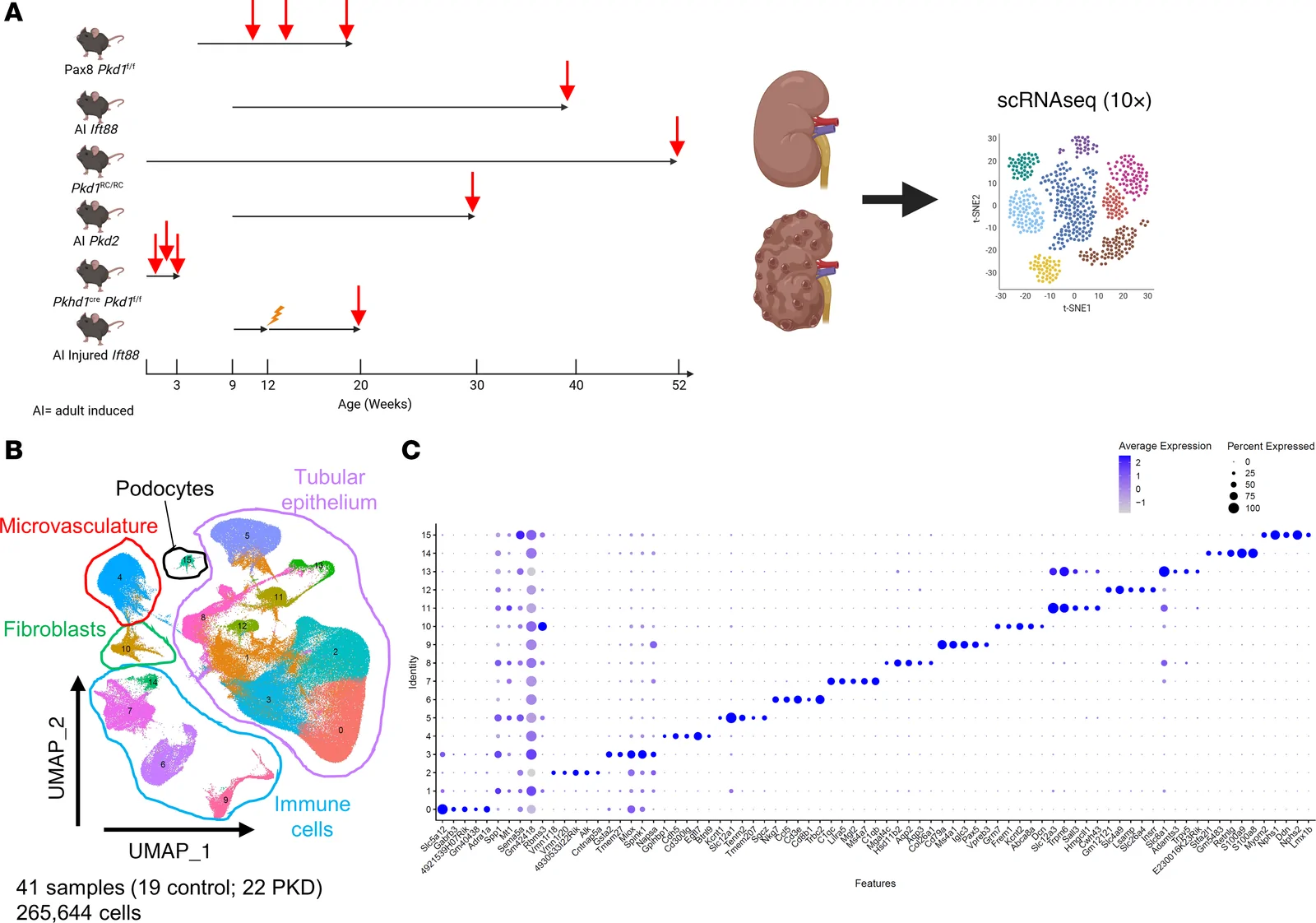
Single-cell atlas of PKD in mice. (**A**) Schematic of experimental design used to generate the single-cell atlas of PKD in mice. Red arrows indicate time points of harvest. (**B**) Uniform Manifold Approximation and Projection (UMAP) of all cells collected from mouse models of PKD. (**C**) Dot plot showing the top 5 markers of each cluster from the whole atlas. Pax8^cre^ *Pkd1^fl/fl^* model (8 control samples, 9 PKD samples; all male), adult induced (AI) *Ift88* model (2 controls, 2 PKD; all female), *Pkd1*^RC/RC^ model (2 controls, 3 PKD; 3 males [1 control, 2 PKD], 2 females [1 control, 1PKD]), AI *Pkd2* (2 controls, 2 PKD; all female), *Pkhd1*^cre^ *Pkd1^fl/fl^* (3 controls, 3 PKD; mix of males and females), AI injured *Ift88* (4 controls [2 sham operated *Ift88,* 2 injured cre negative control], 2 PKD; all female).

We analyzed scRNA-seq or single-nucleus RNA-seq (snRNA-seq) data collected from 6 different mouse models of PKD, including published data — Pax8 tetO^cre^ *Pkd1^fl/fl^* mice harvested 66, 100, or 130 days after doxycycline induction (6); adult induced *Ift88* mice (induced at 8 weeks of age) harvested approximately 7 months after induction (7); adult induced *Pkd2* mice harvested approximately 4 months after induction (8); *Pkhd1*^cre^ *Pkd1^fl/fl^* mice harvested at 7, 14, and 21 days of age (9); and adult induced *Ift88* mice harvested 8 weeks after ischemia reperfusion injury (7) — and unpublished data (*Pkd1*^RC/RC^ mice harvested at 1 year of age; Figure 1A; harvest times depicted with a red arrow). A detailed description of grouping, genetic mutation, cell types impacted by the genetic mutation, time points of harvest, technology used, biological sex, total number of cells analyzed per sample, and PKD severity, when available, can be found in Supplemental Table 1; supplemental material available online with this article; https://doi.org/10.1172/jci.insight.207157DS1 After removal of doublets, ambient RNA, and low-quality cells (see Supplemental Methods), we clustered and annotated 265,644 cells from 41 samples (19 control; 22 PKD), identifying all major cell types including tubular epithelial cells, immune cells, microvasculature, fibroblasts, and podocytes (Figure 1, B and C). Identification and annotation of major cell types was accomplished using cell type–specific marker genes previously identified in the literature (10, 11).

**Figure 2:**
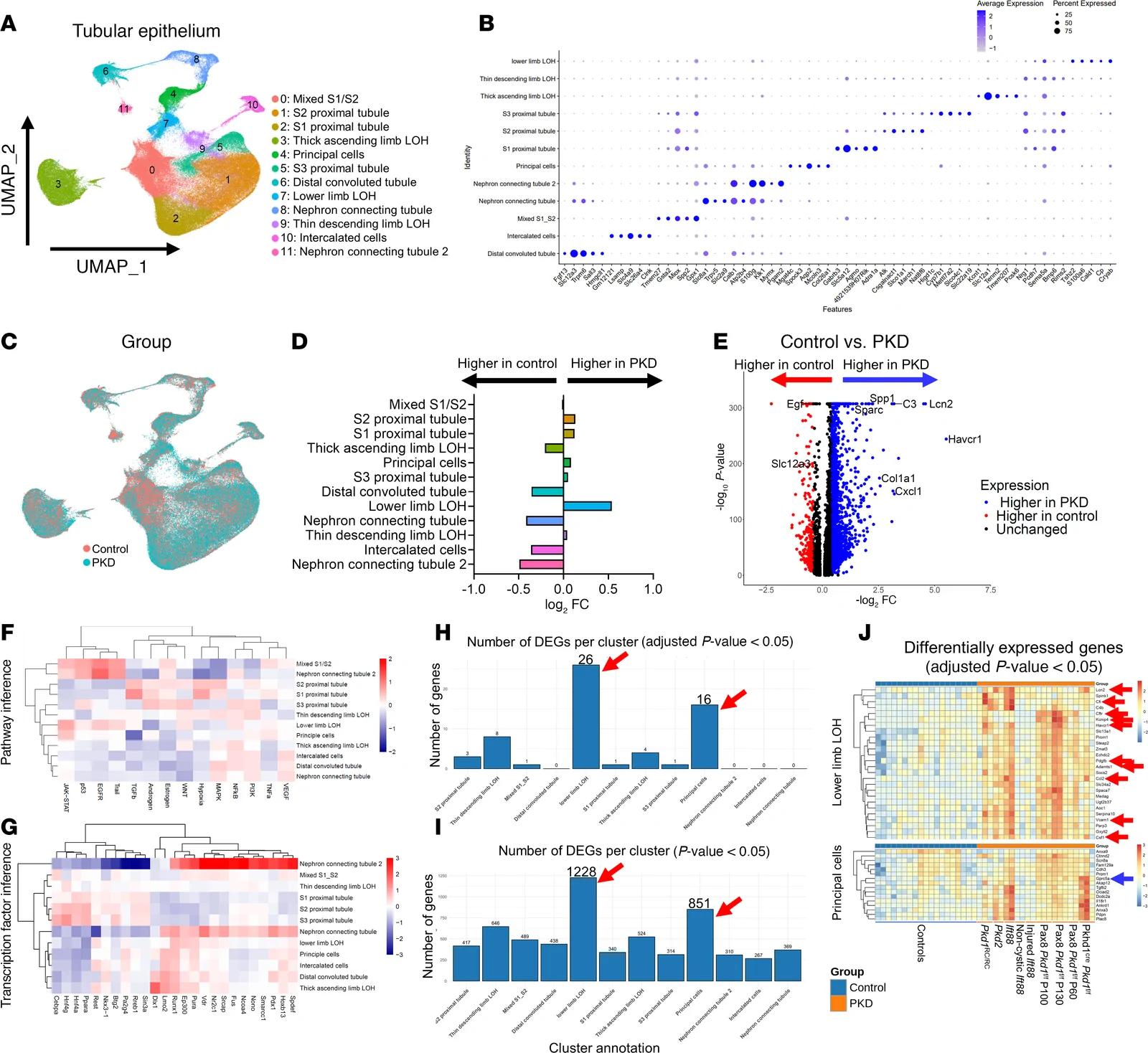
Analyses of scRNA-seq data from the nephron. (**A**) UMAP of tubular epithelial cells subclustered from Figure 1. (**B**) Dot plot showing the top 5 markers of each cluster of cells. (**C**) UMAP of tubular epithelial cells based on experimental group. (**D**) Quantification of cluster abundance in control and PKD samples from whole atlas shown as log_2_(fold change). (**E**) DEGs in all tubular epithelial cells when comparing control and PKD samples. (**F** and **G**) Pathway and transcription factor inference from DecoupleR. (**H** and **I**) Quantification of the number of DEGs in each cluster (control vs. PKD) in pseudobulked scRNA-seq data determined using DESeq2. (**J**) DEGs between control and PKD tubular cell clusters. Wilcoxon rank-sum test (**E** and **H**–**J**).

To better understand how mutations in PKD-related genes impacted tubular epithelium across models, we subsetted epithelial cells from the whole atlas and analyzed them in isolation. Following subsetting, epithelial cells were reclustered to determine what specific subpopulations were present. Analysis of the data revealed 12 transcriptionally distinct clusters of cells spanning the entire length of the nephron, including proximal tubule (PT; clusters 0, 1, 2, and 5), loop of Henle (LOH; clusters 3, 7, and 9), distal tubule (clusters 6, 8, and 11), and collecting duct (clusters 4 and 10; Figure 2, A and B). The proportion of S1–S3 PT, collecting duct principal cells (PCs), lower limb of the LOH, and thin descending limb of the LOH were increased in combined PKD samples compared with controls, with changes in the lower limb LOH being the most substantial (Figure 2, C and D). Across all nephron segments examined, tubular epithelial cells from PKD samples had increased expression of genes associated with tubular injury (*Lcn2* and *Havcr1*), complement (*C3*), inflammation (*Cxcl1*), and fibrosis (*Col1a1*, *Spp1*, and *Sparc*), in agreement with previous data (Figure 2E and Supplemental Table 2) (12, 13). Pathway and transcription factor inference revealed that several clusters expressed genes associated with proinflammatory pathways (JAK-STAT, NF-κB, PI3K, and TNF) and transcription factors (Figure 2, F and G).

To better understand how PKD impacted gene expression in each nephron segment, we performed pseudobulk analysis of our scRNA-seq data, followed by identification of differentially expressed genes (DEGs) in each cluster using DESeq2 (14). This analysis revealed that PCs and cells from the lower limb LOH had the most DEGs when comparing control and PKD samples (Figure 2, H and I), independent of the *P* value threshold that was used (adjusted *P* value < 0.05 or *P* value < 0.05). Further investigation of DEGs (using adjusted *P* value < 0.05) in the lower limb LOH revealed that PKD mice had increased expression of genes associated with kidney injury (*Lcn2*, *Havcr1*, and *Vcam1*), complement (*Cfi* and *C4b*), inflammation (*Csf1* and *Ccl2*), fibrosis (*Pdgfb* and *Admats1*), and fluid/ion secretion (*Cftr* and *Kcnip4*) compared with controls (Figure 2J, red arrows). We also found that expression of *Gprc5a*, a recently identified marker of cyst-lining epithelial cells (6), was increased in PCs from PKD mice (Figure 2J, blue arrow).

When analyzing the data, we noted inconsistencies in gene expression across individual PKD replicates, even within the same disease model. To better understand this variability, we grouped DEGs (adjusted *P* value < 0.05) based on variables that may be driving this effect, including sex, experimental model, time point of harvest, rate of disease progression, and genetic mutation (orthologous vs. non-orthologous) and replotted the data using heatmaps. For rate of disease progression, mice were considered to have rapidly progressing PKD if they developed cysts of any size in 8 weeks or less (*Pkhd1*^cre^ *Pkd1^fl/fl^* model [3 samples]; Injured *Ift88* model [2 samples]). In both the lower limb LOH and PCs, we found that DEGs most strongly segregated based on the rate of disease progression rather than sex, experimental model, time point of harvest, or genetic mutation (Supplemental Figures 1 and 2), suggesting that rate of PKD progression, and not the type of genetic mutation, is the most important variable driving the observed DEGs.

### Individual-model-level comparison of cluster abundance and DEGs in relation to one another and patients with ADPKD.

**Figure 3:**
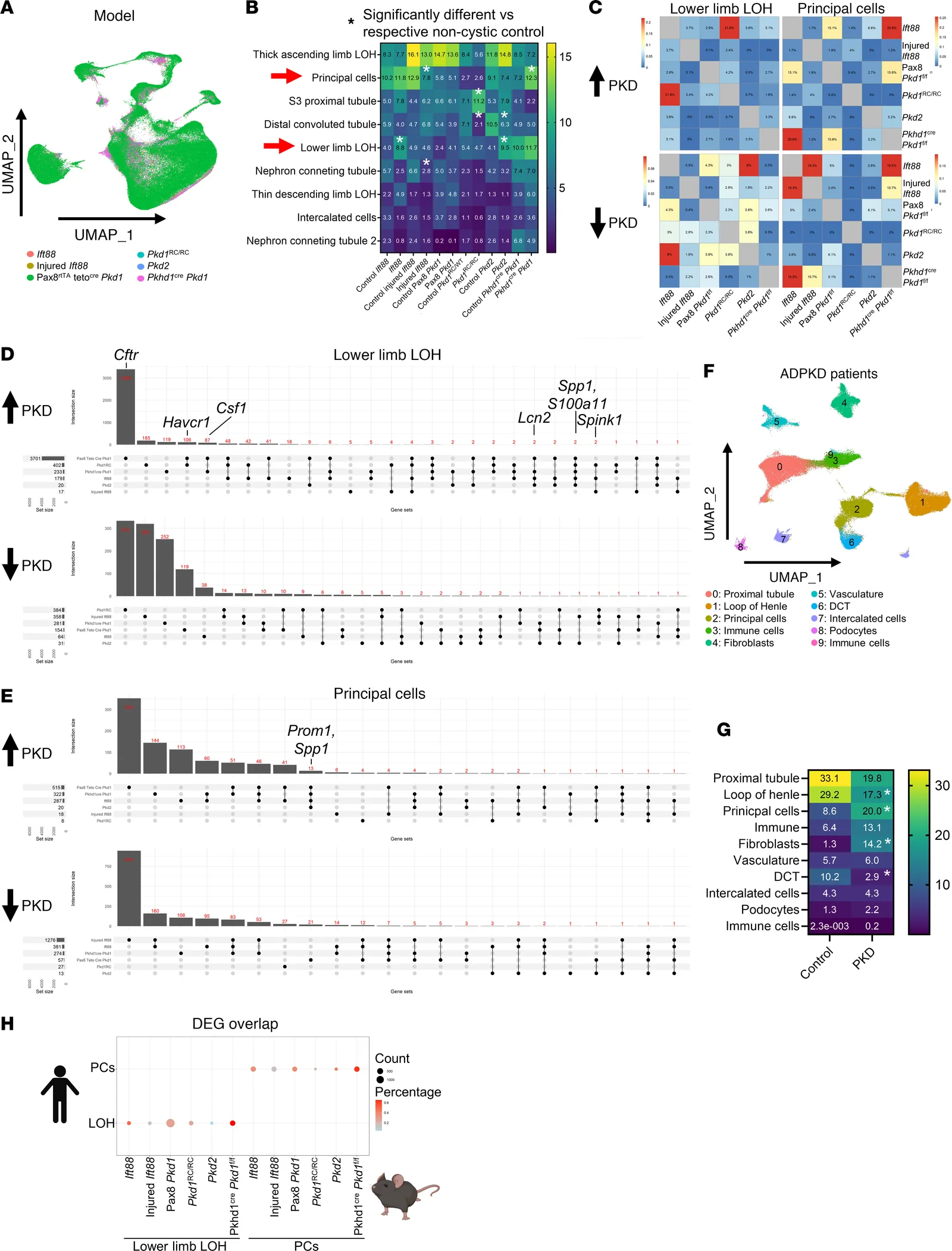
Model-level analysis of cluster abundance and DEGs. (**A**) UMAP of tubular epithelial cells based on individual models. (**B**) Quantification of cluster abundance in individual models. (**C**) Jaccard indices of intersected genes that were increased or decreased in the lower limb LOH and principal cells (PCs) of individual PKD models. (**D** and **E**) Upset plot showing shared genes that are increased or decreased across models in (**D**) lower limb LOH and (**E**) PCs. (**F**) UMAP of control and ADPKD single-cell data. (**G**) Quantification of cluster proportion. (**H**) Dot plot showing the percentage of intersected DEGs when comparing lower limb LOH and PCs between individual mouse models and ADPKD patients. *P < 0.05 by the Wilcoxon rank-sum test (**B** and **G**).

In addition to identifying commonalities in gene expression across PKD mouse models, we also sought to understand model-specific differences in nephron segment abundance and gene expression and how those differences related to patients with ADPKD. As such, we subsetted our atlas at the individual model level and quantified cell proportion and DEGs within each nephron segment (Figure 3A). For this quantification, we excluded samples from the S1 and S2 PT, as we found that the sequencing technology (single cell vs. single nucleus) heavily impacted cluster abundance, independently of phenotype (Supplemental Figure 3). When we analyzed the data, we found that the proportion of PCs, S3 PT, distal convoluted tubule (DCT), lower limb LOH, and nephron connecting tubule was significantly different when comparing control to PKD samples, but only in a few select models (Figure 3B). Of note, we found that the proportion of lower limb LOH cells was increased in the *Ift88* and *Pkd2* models (vs. respective non-cystic controls) and that the proportion of DCT was decreased in *Pkd1*^RC/RC^ and *Pkd2* mice relative to their non-cystic controls (Figure 3B). PCs were increased in the *Pkhd1*^cre^ *Pkd1^fl/fl^* model but decreased in the injured *Ift88* model in relation to non-cystic controls (Figure 3B). The observed differences in cluster abundance occurred despite the fact that the gene of interest is impacted in all nephron segments across PKD models, although some models, such as the *Pkhd1*^cre^ *Pkd1^fl/fl^* model, have greater Cre recombinase activity in the distal nephron (15), which may be reflected by the fact that PCs were the only cluster in this model in which the proportion was different when comparing control and PKD samples.

We next calculated DEGs (adjusted *P* < 0.05) in each nephron segment from each cystic model in relation to non-cystic controls from the same model using the Wilcoxon rank-sum test and plotted the data using Jaccard indices. We focused our analyses on the lower limb LOH and PCs due to the fact that these clusters had the highest number of DEGs at the whole-atlas level. Expectedly, we found that DEG overlap across models was relatively limited, ranging from no overlap to 25.6% of DEGs (Figure 3C). However, quite unexpectedly, we found that the models with the most DEG overlap did not share a common genetic mutation. For example, we found that the *Ift88* and *Pkhd1*^cre^ *Pkd1^fl/fl^* models were most similar in terms of increased DEGs in PCs, while *Ift88* and *Pkd1*^RC/RC^ models were most similar in terms of increased DEGs in the lower limb LOH (Figure 3C). In contrast, the *Ift88* model was most similar to injured *Ift88* and *Pkhd1*^cre^ *Pkd1^fl/fl^* models in terms of decreased DEGs in PCs (Figure 3C). We also visualized intersected genes in these 2 nephron segments using upset plots and found that no genes were increased or decreased in all models, although some genes, such as *Spp1*, were increased in multiple models in both nephron segments (Figure 3, D and E). Thus, while there are certain genes that are enriched in multiple models, each model is unique in terms of DEGs that are altered across the 2 nephron segments analyzed.

Next, we set out to quantify which mouse model was most similar to patients with ADPKD in terms of cluster proportion and gene expression. To do this, we analyzed published snRNA-seq data from patients with ADPKD (16), identifying all nephron segments as well as immune cells, vasculature, and fibroblasts (Figure 3F and Supplemental Figure 4A). Unlike the mouse models analyzed, quantification of human data revealed that patients with ADPKD had a significant decrease in the proportion of cells from the LOH (Figure 3G). The proportion of PCs was increased in patients with ADPKD, similar to what was observed in the *Pkhd1*^cre^ *Pkd1^fl/fl^* model. Patients with ADPKD also had a decreased proportion of DCT cells, similar to what was observed in the *Pkd1*^RC/RC^ and *Pkd2* mouse models (Figure 3G).

Next, we calculated DEGs in each nephron segment of ADPKD kidneys in relation to non-cystic controls using the Wilcoxon rank-sum test and quantified the percentage of overlapping genes in the same nephron segment of each mouse model. In both the lower limb LOH and PCs, the *Pkhd1*^cre^ *Pkd1^fl/fl^* model had the highest frequency of DEG overlap with ADPKD patients while the Pax8 *Pkd1^fl/fl^* had the highest number of intersected genes, likely owing to the fact that this model has the highest number of DEGs when comparing control to PKD samples (Figure 3, D and H). We also found that the *Ift88* model had the second highest frequency of DEG overlap with ADPKD patients in both nephron segments, while the orthologous *Pkd1*^RC/RC^ model ranked near the bottom in terms of DEG overlap with ADPKD patients (Figure 3H). The *Pkhd1*^cre^ *Pkd1^fl/fl^* model had the highest frequency of DEG overlap with ADPKD patients in several other nephron segments, including DCTs, intercalated cells (ICs), and the thin limb LOH (Supplemental Figure 4B). PT DEG overlap with ADPKD patients was similar across most models (Supplemental Figure 4B). It should be noted that the patient samples used in this analysis represent a stage of advanced disease, as is typical in ADPKD patient samples (16). Thus, based on the above data, we conclude that the *Pkhd1*^cre^ *Pkd1^fl/fl^* model is most similar to patients with ADPKD in terms of cluster abundance and DEG overlap at a stage of advanced human disease.

### Identification of PKD-specific clusters in the lower limb LOH and PCs.

**Figure 4:**
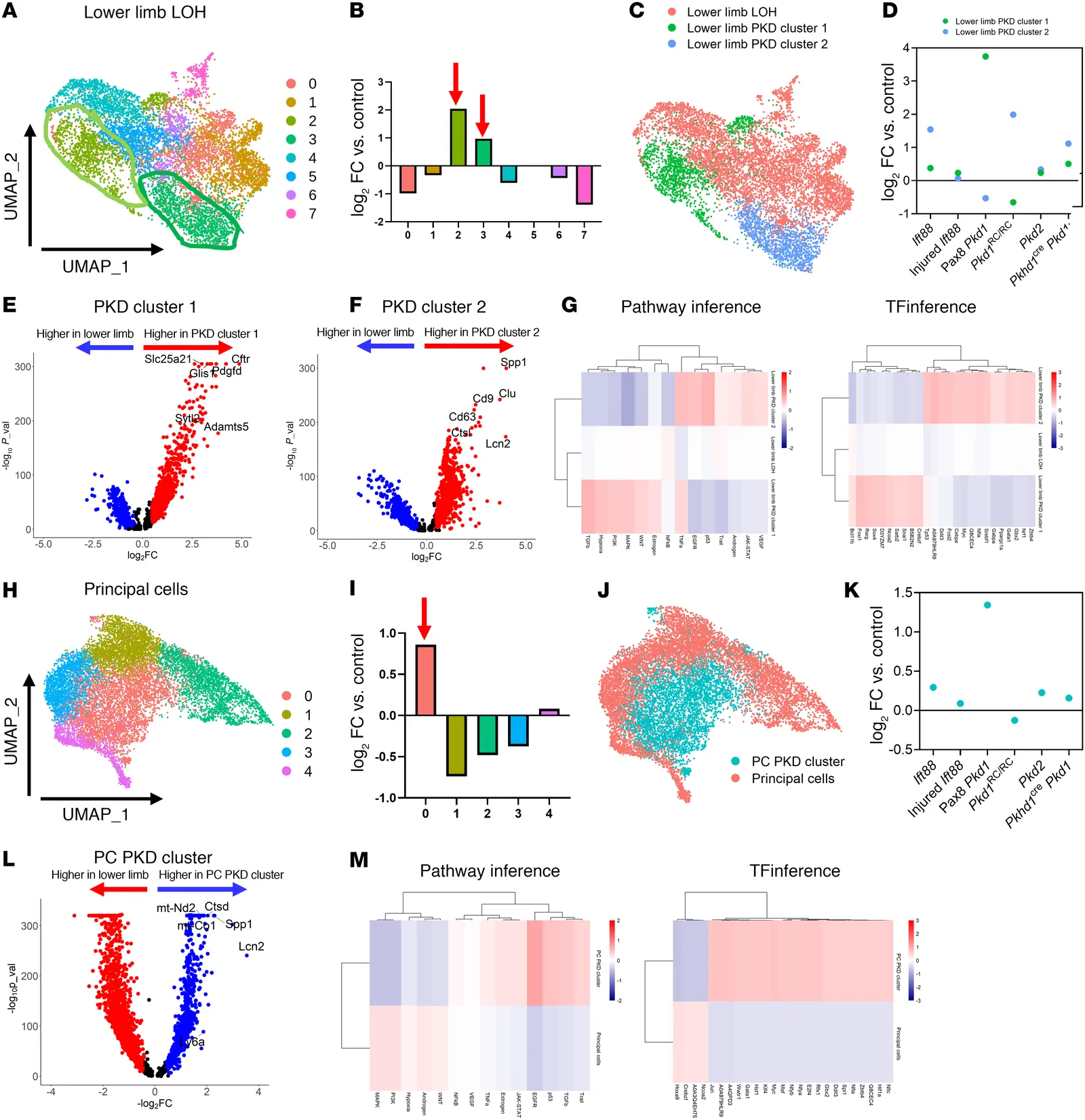
PKD-enriched clusters across mouse models. (**A**) UMAP showing high-resolution clusters and groups in the lower limb LOH. (**B**) Quantification of subsetted, high-resolution lower limb LOH cluster. Two segments were greater than 2-fold increased in PKD samples. (**C**) Re-annotated high-resolution clusters from lower limb LOH. (**D**) Quantification of PKD-enriched cluster abundance in lower limb LOH from individual models in relation to model specific, non-cystic controls. (**E** and **F**) Volcano plot showing DEGs in (**E**) PKD cluster 1 and (**F**) PKD cluster 2 in relation to all other cells in the lower limb LOH. (**G**) Pathway and transcription factor inference of genes enriched in PKD cluster 1 and PKD cluster 2. (**H**) UMAP showing high-resolution clusters and groups in principal cells (PCs). (**I**) Quantification of subsetted, high-resolution PC cluster. One segment was greater than 2-fold increased in PKD samples versus non-cystic controls. (**J**) Re-annotated high-resolution clusters from PCs. (**K**) Quantification of PKD-enriched cluster abundance in PCs from individual models in relation to model specific, non-cystic controls. (**L**) Volcano plot showing DEGs in PC PKD cluster in relation to all other PCs. (**M**) Pathway and transcription factor inference of genes enriched in the PC PKD cluster.

In our initial clustering, we grouped all cells from each nephron segment into one homogeneous population, without making assumptions about PKD-specific subsets that may be embedded within these nephron segments. To determine whether there were PKD-enriched subsets embedded within each nephron segment, we subsetted, reclustered, and quantified the abundance of each nephron segment at high resolution (Supplemental Figure 5A). We focused our analysis on the lower limb LOH and PCs due to the fact that these nephron segments were the most impacted in PKD samples, both at the whole-atlas and individual-model level. This analysis revealed that the lower limb LOH had 2 subsetted clusters that were greater than 2-fold enriched in PKD samples relative to non-cystic controls (Figure 4, A and B). These segments, which we annotated as lower limb PKD cluster 1 and lower limb PKD cluster 2, had highly disparate enrichment among individual PKD models (Figure 4, C and D). For example, lower limb PKD cluster 1 was almost exclusively derived from the Pax8 *Pkd1^fl/fl^* model, while lower limb PKD cluster 2 was derived from the *Ift88*, *Pkd1*^RC/RC^, and *Pkhd1*^cre^ *Pkd1^fl/fl^* models (Figure 4D). DEGs in each of the PKD-enriched clusters from the lower limb were unique (Figure 4, E and F). Pathway and transcription factor inference supported this claim, showing that lower limb PKD cluster 1 had an enrichment of genes associated with the TGF-β and WNT pathways, whereas lower limb PKD cluster 2 had an enrichment of genes associated with EGFR, p53, and VEGF pathways (Figure 4G). Both PKD clusters were enriched for TNF-α pathway genes.

Analysis of subsetted PCs revealed that a single cluster, cluster 0, was enriched in combined PKD samples compared with controls (Figure 4, H–J). Enrichment of this cluster in PKD samples was largely driven by the Pax8 *Pkd1^fl/fl^* model, although several other models also had increased numbers in PKD samples compared with controls (Figure 4K). The PC PKD cluster had enrichment of mitochondrial genes as well as *Spp1* and *Lcn2* (Figure 4L). Pathways enriched in the PC PKD cluster include EGFR, p53, TGF-β, TNF-α, and JAK-STAT (Figure 4M). In contrast to previous data (6), we did not find an obvious PKD-enriched cluster in PTs, although we did find 2 PKD-enriched clusters in the thin descending limb LOH (Supplemental Figure 5, B and C).

### Atlas- and model-level analyses of cell-cell communication show consistent enrichment of SPP1 signaling in PKD clusters.

Our data indicate that the lower limb LOH and PCs are the nephron segments most altered in PKD. Furthermore, by subsetting and reclustering at high resolution, we were able to identify 3 PKD-specific clusters (lower limb PKD cluster 1, lower limb PKD cluster 2, and the PC PKD cluster) in the combined segments. To understand whole-atlas- and model-level signaling between PKD-specific clusters and other cell types, we performed CellChat on the fully annotated single-cell atlas (Supplemental Figure 6). In the whole atlas, we found that the number and strength of interactions were increased between lower limb PKD cluster 1 and fibroblasts in PKD samples (Supplemental Figure 7A). The strength, albeit not the number, of interactions was also increased in PKD samples from lower limb PKD cluster 2 and PC PKD cluster in the whole atlas (Supplemental Figure 7A). When we analyzed signaling interactions at the model level, we found that the number and strength of interactions were highest in lower limb PKD cluster 1 in the majority of models, with the exception of the injured *Ift88* and *Pkhd1*^cre^ *Pkd1^fl/fl^* models (Supplemental Figure 7, B–G). Of interest, we noted that the number and strength of signaling interactions were most frequently increased between lower limb PKD cluster 1, immune cells, and fibroblasts (Supplemental Figure 7, B–G).

**Figure 5:**
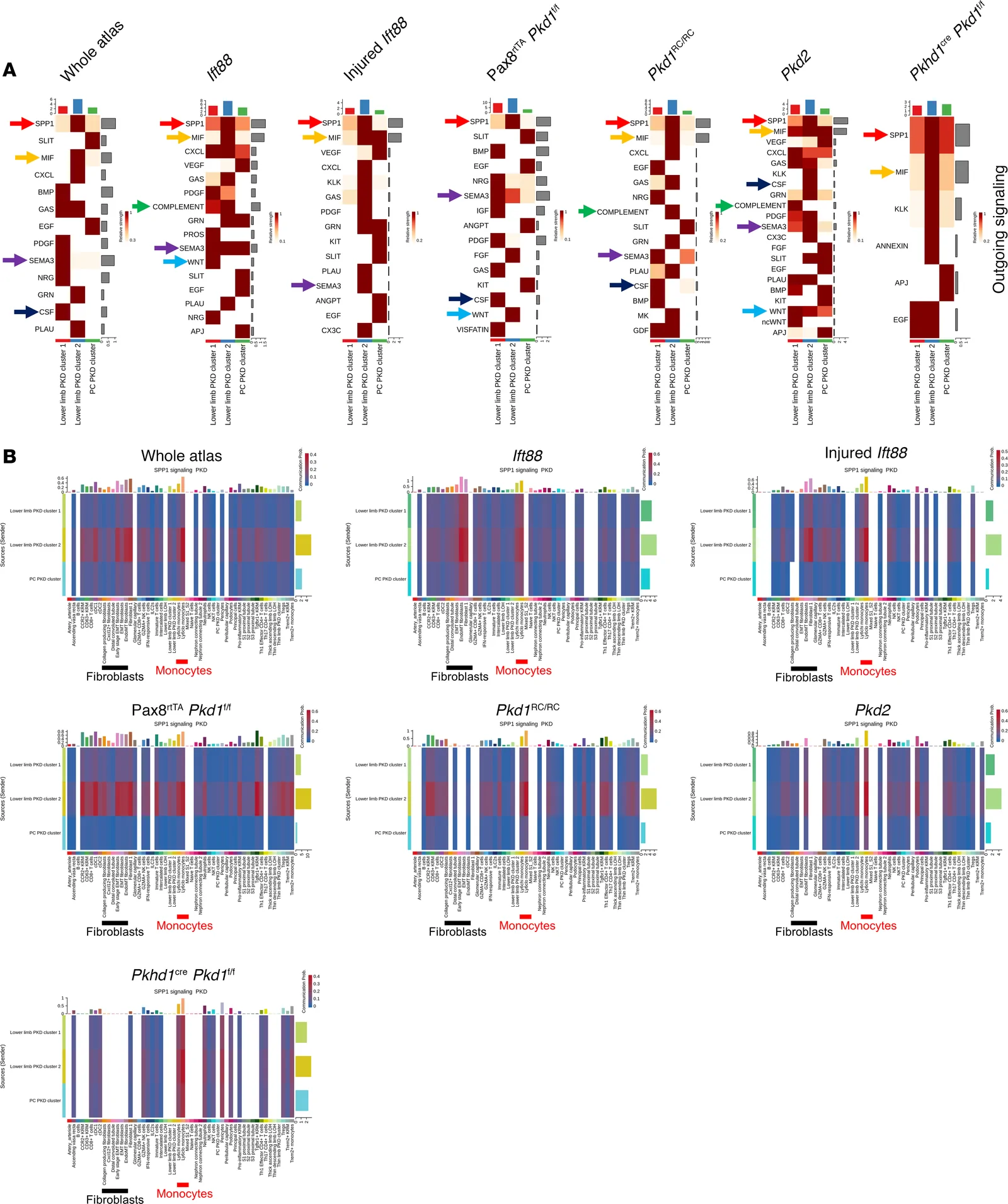
CellChat analysis of cell-cell communication in PKD-enriched clusters at the whole-atlas and model level reveals elevated outgoing SPP1 signaling. (**A**) Top outgoing signaling pathways from PKD-enriched clusters at the whole-atlas or model level. Arrows indicate pathways that are conserved across multiple models. (**B**) SPP1 signaling from PKD-enriched clusters to all other cell types at the whole-atlas or model level.

We next analyzed outgoing signaling from PKD-enriched clusters at the atlas and model level. Analysis of the data revealed that SPP1 was the strongest outgoing signaling pattern in the whole atlas and in each individual model (red arrows) (Figure 5A). With regards to cell types, we found that lower limb PKD cluster 2 had the highest outgoing signaling strength across models (Figure 5A). Other outgoing signaling pathways of interest that were shared in multiple models included complement (green arrows), CSF (dark blue arrows), SEMA3 (purple arrows), WNT (light blue arrows), and MIF (yellow arrows). These pathways along with SPP1 signaling have been associated with PKD in the past, confirming the robustness of our analysis (17–22).

When we analyzed incoming pathways to the PKD-specific clusters, we once again found that SPP1 was the top pathway (Supplemental Figure 8). Incoming WNT and MIF signaling was also observed in multiple models. Other pathways of interest shared across multiple models included galectin (orange arrows), TGF-β (black arrows), IGF (pink arrows), FGF (light green arrows), MK (gray arrows), and TWEAK (salmon arrows), several of which have also been associated with PKD (Supplemental Figure 8) (21, 23–26). These data suggest that PKD-enriched clusters receive signaling from multiple growth factor signaling pathways, which may alter cyst growth.

We next analyzed SPP1 signaling from the PKD clusters, as this pathway was the most consistently enriched outgoing pathway across PKD models. The data indicate that SPP1 from PKD-enriched clusters, particularly lower limb PKD cluster 2, most frequently interacted with fibroblasts and monocytes across models (Figure 5B). A more detailed analyses of SPP1 signaling from lower limb PKD cluster 2 to fibroblasts and monocytes revealed distinct ligand-receptor pairs in each model (Supplemental Figures 9–12). However, we did note consistent Spp1^–^Itgα4^+^Itgβ1 and Spp1^–^Cd44 signaling between lower limb PKD cluster 2 and monocytes across models (Supplemental Figures 9–12), in line with data indicating that SPP1 serves as a monocyte chemoattractant (27, 28). The Spp1^–^Cd44 signaling axis in particular has also been implicated in kidney injury models as a key pathway involved in neutrophil recruitment and impaired tubular regeneration (29). Across models we also observed consistent signaling between neutrophils and lower limb PKD cluster 2, suggesting the pathway may function in a similar manner in PKD (Supplemental Figures 9–12).

### Loss of Spp1 improves PKD in the orthologous Pkd1^RC/RC^ model.

**Figure 6:**
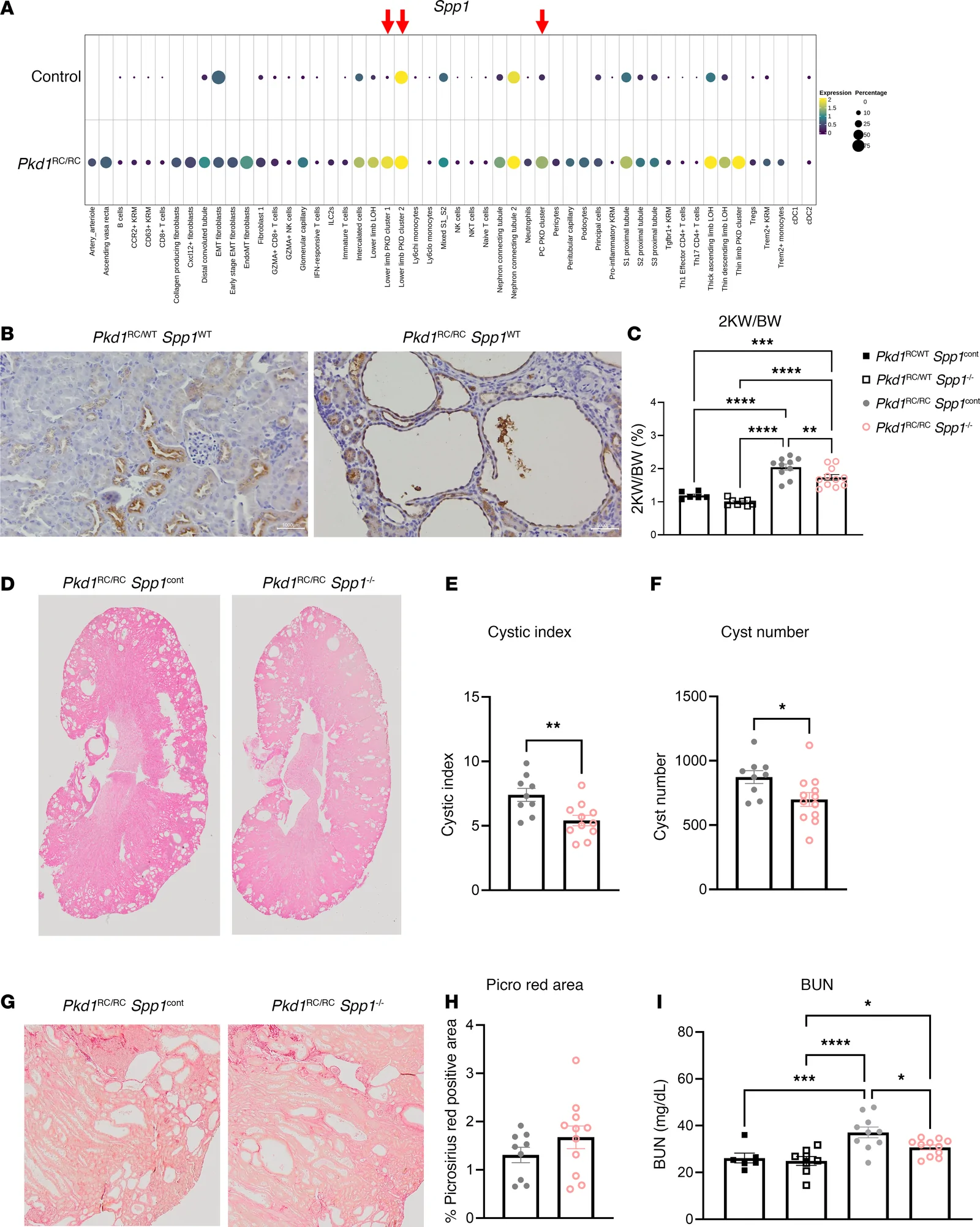
Loss of *Spp1* improves PKD severity in the *Pkd1*^RC/RC^ model. (**A**) Dot plot showing expression of *Spp1* in all cell types of *Pkd1*^RC/RC^ mice. (**B**) Immunohistochemistry staining of SPP1 (osteopontin) in *Pkd1*^RC/WT^ and *Pkd1*^RC/RC^ sections. Original magnification, ×20. (**C**) Quantification of 2 kidney weight to body weight (2KW/BW) ratio in *Pkd1*^RC/WT^ *Spp1*^cont^, *Pkd1*^RC/WT^ *Spp1*^–/–^, *Pkd1*^RC/RC^ *Spp1*^cont^, and *Pkd1*^RC/RC^ *Spp1*^–/–^ mice at 1 year of age. (**D**–**F**) H&E-stained images of *Pkd1*^RC/RC^ *Spp1*^cont^, and *Pkd1*^RC/RC^ *Spp1*^–/–^ mice along with quantification of (**E**) cystic index and (**F**) cyst number. (**G** and **H**) Picrosirius red–stained sections (**G**) and quantification (**H**). (**I**) Quantification of kidney function as measured by blood urea nitrogen (BUN). *P < 0.05; **P < 0.01; ***P < 0.001; ****P < 0.0001 by 1-way ANOVA (**B** and **I**) or Student’s *t* test (all others). *Pkd1*^RC/WT^ *Spp1*^cont^ = 6 mice, *Pkd1*^RC/WT^ *Spp1*^–/–^ = 8 mice, *Pkd1*^RC/RC^ *Spp1*^cont^ = 10 mice, *Pkd1*^RC/RC^ *Spp1*^–/–^ = 12 mice; 36 total *Pkd1*^RC/RC^ mice.

Analysis of scRNA-seq data indicated that SPP1 gene expression and signaling is enriched in PKD enriched clusters and signals to monocytes and fibroblasts. In order to investigate the functional importance of SPP1 signaling in mouse models of PKD, we crossed the orthologous *Pkd1*^RC/RC^ model to commercially available *Spp1*-knockout mice and analyzed the PKD phenotype at 1 year of age. We chose this model due to its orthologous nature and the fact that the rate of PKD progression mimics that of patients with ADPKD (30). We first confirmed that *Spp1* expression was strongly increased in PKD-enriched clusters from *Pkd1*^RC/RC^ scRNA-seq data (Figure 6A, red arrows) and was also present in cyst-lining cells of *Pkd1*^RC/RC^ mice at the protein level (Figure 6B). Analyses of 1-year-old *Pkd1*^RC/RC^ mice showed that loss of *Spp1* modestly reduced 2KW/BW, cystic index, and cyst number compared with age-matched, littermate controls (Figure 6, C–F). Despite the fact that no significant differences were found in fibrosis between groups as indicated by Picrosirius red staining, kidney function was improved in *Pkd1*^RC/RC^ *Spp1*-knockout mice compared with *Pkd1*^RC/RC^ *Spp1* control mice (Figure 6, G–I). Loss of *Spp1* did not impact Ly6c^hi^ monocyte numbers but reduced the number of Ly6c^lo^ monocytes in *Pkd1*^RC/RC^ mice, while other immune cell numbers remained unchanged (Supplemental Figure 13). Whether the loss of *Spp1* and corresponding improvement in PKD severity is due to reduced SPP1-mediated Ly6c^lo^ monocyte recruitment is unknown.

### Validation of enriched SPP1 expression and signaling in ADPKD tissue.

**Figure 7:**
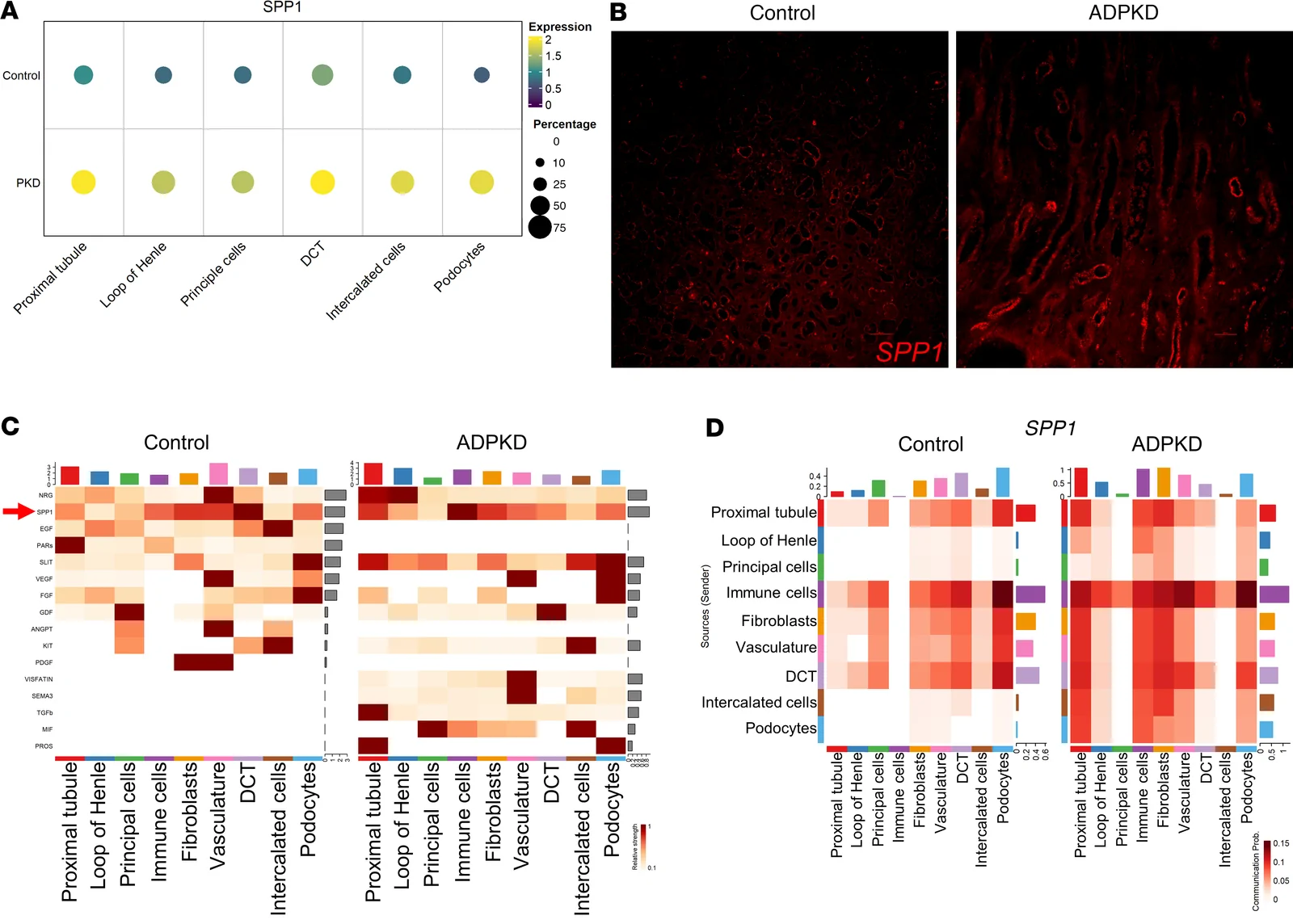
SPP1 expression and signaling are enriched in patients with ADPKD. (**A**) Dot plot showing expression of *SPP1* in nephron segments from control and ADPKD patients. (**B**) RNA scope images showing *SPP1* expression in control and ADPKD tissue. (**C**) Heatmap showing top outgoing signaling pathways in each cell type from control and ADPKD scRNA-seq data. (**D**) SPP1 signaling in control and ADPKD clusters.

To validate the therapeutic potential of targeting *Spp1* in patients with ADPKD, we first analyzed expression of *SPP1* in scRNA-seq data collected from control and ADPKD tissue. The data indicate that *SPP1* expression was increased across nephron segments, including LOHs and PCs (Figure 7A). We also found that *SPP1* expression was increased, albeit not specific to cyst-lining epithelia, in ADPKD kidney tissue compared with control tissue (Figure 7B). Next, we performed CellChat analysis on scRNA-seq data from control and ADPKD kidney tissue. Analysis of the data indicated that SPP1 signaling is the second strongest outgoing signaling pathway in ADPKD kidney tissue (Figure 7C). Similar to what was observed in animal models, SPP1 produced by tubular cells predominantly signaled to immune cells and fibroblasts (Figure 7D). Collectively, these data indicate that *SPP1* expression and signaling is enriched in ADPKD kidney tissue.

## Discussion

In this study, we generated a comprehensive scRNA-seq atlas comprised of 6 different mouse models of PKD, whereby we provide a detailed description of genes, pathways, and signaling interactions that are disrupted across and within individual PKD models and cell types. Using the integrated atlas, we found that the proportion and gene expression of PCs and lower limb LOH were most consistently altered across PKD mouse models. By cross-referencing DEGs from mouse models with ADPKD patients, we found that the *Pkhd1*^cre^ *Pkd1^fl/fl^* model was most similar to ADPKD patients as measured by cluster abundance and DEG overlap. By subsetting and reclustering nephron segments at high resolution, we also identified 3 specific PKD-enriched clusters that were embedded within PCs and lower limb LOH. Analysis of cell-cell communication shows that SPP1 signaling was the strongest outgoing signaling pathway from PKD-enriched clusters across all mouse models analyzed, and signaled to monocytes and fibroblasts. Global deletion of *Spp1* in the orthologous *Pkd1*^RC/RC^ model modestly reduced cystic severity and led to a slight improvement in kidney function. Lastly, we validated enriched SPP1 expression and signaling in ADPKD patient tissue. To facilitate the use of these data among the PKD community, we developed a user-friendly and freely accessible website (https://bmblx.bmi.osumc.edu/scPKD/), whereby researchers can query genes of interest in the whole scRNA-seq atlas or in individual mouse models. We have also provided a visual step-by-step guide for navigating the website in Supplemental Figure 14.

In our study, we compared DEGs (control vs. PKD) identified in each nephron segment from each individual mouse model with DEGs from the corresponding nephron segment in humans. When we did this analysis for the lower limb LOH and PCs, we found that the *Pkhd1*^cre^ *Pkd1^fl/fl^* model had the highest percentage of DEG overlap. Quite surprisingly, we found that the non-orthologous *Ift88* model shared the second highest DEG overlap with ADPKD patients, while the orthologous *Pkd1*^RC/RC^ model showed modest DEG overlap with ADPKD patients in both nephron segments. A similar phenomenon was observed when analyzing other distal nephron segments (DCT, ICs, thick ascending limb LOH, thin limb LOH), although these differences appeared to dissipate when analyzing PT segments. Although uncertain, the strong overlap between the *Pkhd1*^cre^ *Pkd1^fl/fl^* model and ADPKD patients may be driven by the fact that data from both groups were obtained at a stage of advanced disease. The striking finding that the *Ift88* model shared the second highest DEG overlap with ADPKD patients while the *Pkd1*^RC/RC^ model performed relatively poorly strongly suggests that the type of genetic mutation is not the most important factor when determining which animal model to select, despite the fact that orthologous models are routinely thought of as being more appropriate in preclinical studies (30). It is important to note that the *Ift88* and *Pkd1*^RC/RC^ mice had a nearly identical PKD phenotype at the time of analysis, suggesting that factors such as the microenvironment or disease kinetics can impact these models’ similarity to ADPKD patients.

In our model, we consistently noted that *Spp1* expression and signaling was enriched in PKD samples. Furthermore, we found that SPP1 predominantly signaled to monocytes and fibroblasts in all models and ADPKD patients, suggesting that enriched *Spp1* expression and signaling may be a unifying feature of PKD mouse models and patients. These results are similar to reported findings in other fibrotic renal diseases, including chronic kidney disease, acute kidney injury, idiopathic membranous nephropathy, glomerulosclerosis, and hydronephrosis, where SPP1/fibroblast signaling and enriched *Spp1* expression have been found (29, 31–34). This shared SPP1 phenotype suggests that increased *Spp1* expression may be a hallmark of damaged or fibrotic epithelial cells rather than a unique feature of any one renal disease, and support its potential as a future therapeutic target across fibrotic renal diseases. Overall, our results add to a body of evidence showing a contribution of SPP1 to fibrotic kidney diseases and support its use as a biomarker of renal dysfunction.

Standard recommendations for the field in selecting preclinical study models emphasize choosing orthologous models with a slower disease progression, which mimics human disease (30). In the current study, we used the orthologous *Pkd1*^RC/RC^ model to determine the impact of *Spp1* knockout on cystic disease. Of note, when we globally deleted *Spp1*, we found that cyst severity and kidney function were significantly improved, which is in contrast to a recent publication from our group showing that loss of *Spp1* improves cyst severity but worsens fibrosis in the *pcy/pcy* mouse (35). Since our scRNA-seq atlas suggests that enriched SPP1 expression and signaling is uniform across models, the reason for the phenotypic discrepancy upon *Spp1* global deletion in the *Pkd1*^RC/RC^ and *pcy/pcy* models is uncertain. While global *Spp1* deletion in the absence of cystic disease does not lead to any developmental or phenotypic abnormalities in mice, collagen deposition and organization was found to be altered during wound healing (36). It is that possible during cystic disease there is a difference in ECM architecture in the *Spp1*-knockout animals and this difference functions in a unique way within the context of the model’s genetic mutation. Furthermore, the cystic index observed in the *pcy/pcy* mice was approximately double that observed in our *Pkd1*^RC/RC^ animals, suggesting the stage of disease at which *Spp1* global deletion is assessed could also contribute to the differences we observed between the two studies. Additional studies at different stages of disease and across genetic models are needed to clarify the role of *Spp1* in PKD. Additionally, given the relatively poor DEG similarity between human patients and the *Pkd1*^RC/RC^ model found in these studies, future investigators evaluating the role of SPP1 should utilize a model with increased similarity to ADPKD patients. Despite the ambiguity in function, the uniformity and prevalence of SPP1 expression and signaling found in our scRNA-seq atlas and its presence in ADPKD patients strongly support further exploration of this signaling axis to determine whether it presents a valid therapeutic target. Moreover, our findings highlight that even when scRNA-seq data predict that a common alteration is present across multiple PKD models, functional validation is still required.

In conclusion, we generated an integrated single-cell atlas of cystic kidney disease across multiple PKD mouse models. Using this atlas, we identified dysregulated SPP1 signaling that was conserved across mouse models and show that global loss of *Spp1* modestly reduces cystic severity in the orthologous *Pkd1*^RC/RC^ model.

## Methods

### Sex as a biological variable.

These studies contain data from both male and female animals, and similar findings were found in both sexes.

### scRNA-seq.

This study combined previously published scRNA-seq data (6, 8, 9, 16) with new data as outlined in Supplemental Methods. After sequencing, data were aligned to the reference genome (mm10) using Cellranger (10X Genomics). The resulting matrix files were loaded into Seurat version 5.2.0 followed by removal of doublets (DoubletFinder) (37) and ambient RNA (Soupx) (38). Individual samples were merged using the Merge function in Seurat followed by data integration using SCT-RPCA and Harmony (39). Cells with over 50% of genes mapping to mitochondrial transcripts and unique gene counts greater than 3,000 or less than 200 were discarded, thresholds that have previously been established for kidney scRNA-seq data (7). After quality control measures and integration, we followed the standard Seurat vignette for clustering and data visualization. To identify DEGs in each cell cluster, we used the FindAllMarkers function in Seurat on the normalized gene expression data. To find differences between control and PKD samples, we used the Wilcoxon rank-sum text and FindMarkers function in Seurat or DESeq2 (14) on data that was pseudobulked using the AggregrateExpression function and the counts slot. Cell-cell communication was analyzed using CellChat (40), while pathway and transcription factor inference was done using decoupleR (41).

### Statistics.

Statistical significance was determined using the Wilcoxon rank-sum test, 1-way analysis-of-variance (ANOVA) without correction for multiple comparisons, or a 2-tailed unpaired Student’s *t* test when applicable. Residual plots were used to determine normality of the data. Analysis was performed using Seurat v5 (Wilcoxon rank-sum test) and GraphPad Prism v11 (1-way ANOVA, *t* test). A *P* value of less than 0.05 was considered significant.

### Study approval.

All animals were maintained in Association for Assessment and Accreditation of Laboratory Animal Care (AALAC) International–accredited facilities in accordance with Institutional Animal Care and Use Committee regulations at the University of Oklahoma Health Sciences Center under approved protocol number 23-002-CHIX.

### Data availability.

All raw scRNA-seq data are available in the NCBI GEO as outlined in Supplemental Methods. Additional metadata information and supporting data can be found in processed and annotated.rds files for the whole atlas, and each individual model can be downloaded from the website (https://bmblx.bmi.osumc.edu/scPKD/). Code used to generate figures is available in the lab’s GitHub account (kzimmer1) under the repository name “Mouse-SingleCell-Atlas.” Individual values for all data points shown can be found in the Supporting Data Values file.

## Author contributions

KAZ, SJM, DAL, and DJJ were responsible for designing research studies. SJM, JRG, AMC, MEY, AY, MEH, YP, FN, and BM conducted experiments. SJM, HZ, CNZ, CID, VD, AM, YP, FN, BM, SL, Xiaoyan Li, JRG, Xiaogang Li, WW, ZL, MCMWE, BKY, BDC, KH, JRS, and QM contributed to acquiring and analyzing data. SJM and KAZ contributed to writing the manuscript, KAZ, SJM, DJJ, and DAL were responsible for funding the above studies.

## Conflict of interest

The authors have declared that no conflict of interest exists.

## Funding support

This work is the result of NIH funding, in whole or in part, and is subject to the NIH Public Access Policy. Through acceptance of this federal funding, the NIH has been given a right to make the work publicly available in PubMed Central.

- NIH grants K01DK119375 (to KAZ), 1K01DK144648 (to SJM), 2R01DK122212 (to JRS and KAZ), R01DK115752 (to BKY), R01DK129255 (to KAZ), 1R21DK140693 (to KAZ), and 1L30-DK137350 (to SJM).
- Polycystic Kidney Disease Research Foundation grants 826369 (to KAZ) and 1065555 (to SJM).
- Presbyterian Health Foundation seed grant (to KAZ).
- Wellcome Trust Accelerator Award 314710/Z/24/Z (to DJJ) and Wellcome Trust Investigator Award 220895/Z/20/Z (to DAL).
- Specialised Foundation Programme at the East of England NHS Deanery (to DJJ).
- NIHR Biomedical Research Centre at Great Ormond Street Hospital for Children NHS Foundation Trust and University College London (to DAL).
- NIH grants P30-DK079337 (to the UAB-UCSD O’Brien Center for Acute Kidney Injury Research), P30-AR048311 and P30-AI27667 (to the UAB Comprehensive Flow Cytometry Core), and 1S10OD028479-01 (to the Oklahoma Medical Research Foundation core facility for purchase of the Aurora Cytek).

## Supplementary Material

Supplemental data

Supplemental table 1

Supplemental table 2

Supplemental table 3

Supplemental table 4

Supplemental table 5

Supporting data values

## Version 1 07/28/2026

In-Press Preview

## Version 2 09/08/2026

Electronic publication
