# Supplementary material for: A cross-model single-cell atlas reveals conserved involvement of osteopontin in polycystic kidney disease
Source: JCI Insight. 2026 Jul 28;11(17):e207157. doi: 10.1172/jci.insight.207157 (PMC13564071; doi:10.1172/jci.insight.207157)
Supplement: Supplementary file 1 [file jciinsight-11-207157-s043.pdf]

Supplemental Figures

Supplemental figure 1. Heatmaps, grouped by metadata variables, showing genes that were significantly different (adjusted p value < 0.05) in the lower limb LOH from control and PKD samples. *Heatmap of DEGs in lower limb LOH between control and PKD samples based on* **(A)** *sex,* **(B)** *PKD model,* **(C)** *day harvested,* **(D)** *rate of PKD progression, and* **(E)** *genetic mutation. Color intensity in each heat map represents each DEGs standard deviation from the mean expression, calculated by* log_2 *transformation of normalized gene counts.*

Supplemental figure 2. Heatmaps, grouped by metadata variables, showing genes that were significantly different (adjusted p value < 0.05) in principal cells from control and PKD samples. *Heatmap of DEGs in principal cells between control and PKD samples based on* **(A)** *sex,* **(B)** *PKD model,* **(C)** *day harvested,* **(D)** *rate of PKD progression, and* **(E)** *genetic mutation. Color intensity in each heat map represents each DEGs standard deviation from the mean expression, calculated by* log_2 *transformation of normalized gene counts.*

Supplemental figure 3. Technology-specific effects on cluster abundance in the proximal tubule. (A) *UMAP showing tubular epithelial cell clustering based on the technology (single cell vs single nucleus).* **(B)** *Quantification of proximal tubule cluster abundance in each model by technology.*

Supplemental figure 4. Comparison of DEGs in each nephron segment between mouse and humans. (A) *Dotplot showing the top 5 marker genes of each cluster from human ADPKD*

single cell data. **(B)** *Dotplot showing the percentage of intersected DEGs (control vs PKD) when comparing nephron clusters between individual mouse models and ADPKD patients.*

Supplemental figure 5. Analysis of subsetted high resolution clusters. (A) *Quantification of subsetted, high resolution cluster abundance in each nephron segment. Data is plotted as Log_2FC in relation to non-cystic controls.* **(B)** *UMAP showing high resolution cluster of thin descending limb LOH.* **(C)** *Quantification of subsetted, high resolution thin limb LOH cluster. Two segments were greater than two-fold increased in PKD samples.*

Supplemental figure 6. Fully annotated scRNAseq atlas of mouse PKD. *UMAP showing the fully annotated scRNAseq atlas of mouse PKD.*

Supplemental figure 7. Signaling interactions between PKD-enriched cluster and all other cell types at the whole atlas and individual model level. (A-G) *Number and strength of interactions between PKD enriched clusters and all other cell types in the* **(A)** *combined atlas and* **(B-G)** *each individual model. Red indicates that the communication between the two cell types is increased in PKD while blue means it is decreased in PKD.*

Supplemental figure 8. Incoming signaling pathways to PKD-enriched clusters. *Heatmaps showing incoming signaling pathways to PKD-enriched clusters. Arrows indicate pathways that are conserved across models.*

Supplemental figure 9. Dotplot showing ligand receptor pairs between lower limb PKD cluster 2 and other cell types in the kidney in scRNAseq data. *Dotplot showing ligand-receptor pairs between lower limb PKD cluster 2 and all other cells types in* **(A)** *the whole atlas or* **(B)** *Ift88 model.*

Supplemental figure 10. Dotplot showing ligand receptor pairs between lower limb PKD cluster 2 and other cell types in the kidney in scRNAseq data. *Dotplot showing ligand-receptor pairs between lower limb PKD cluster 2 and all other cells types in* **(A)** *the injured Ift88 or* **(B)** *Pax8 Pkd1*$^{f/f}$ *model.*

Supplemental figure 11. Dotplot showing ligand receptor pairs between lower limb PKD cluster 2 and other cell types in the kidney in scRNAseq data. *Dotplot showing ligand-receptor pairs between lower limb PKD cluster 2 and all other cells types in* **(A)** *the Pkd1*$^{RC/RC}$ *or* **(B)** *Pkd2 model.*

Supplemental figure 12. Dotplot showing ligand receptor pairs between lower limb PKD cluster 2 and other cell types in the kidney in scRNAseq data. *Dotplot showing ligand-receptor pairs between lower limb PKD cluster 2 and all other cells types in Pkhd1*^{cre} *Pkd1*$^{f/f}$ *model.*

Supplemental figure 13. Immune cell numbers in *Pkd1*$^{RC/RC}$ mice with and without *Spp1*. *Flow cytometry quantification of immune cell numbers in control, Pkd1*$^{RC/RC}$ *Spp1*^{WT}*, Pkd1*$^{RC/RC}$ *Spp1-/- mice harvested at one year of age.*

Supplemental figure 14. Visual step-by-step guide to navigate the single cell website.

A. Home page display includes links to individual datasets, single-cell ATLAS visualization, dataset downloads, as well as help and feedback. The home page also displays dataset details split by species and sex. B. The ATLAS web page features a large UMAP display of selected ATLAS (human or mouse), which can then be filtered by cell type, sex, model (mouse ATLAS only), or disease status. Gene expression can be visualized for selected and filtered ATLAS, and is displayed both overlaid onto a UMAP as well as across cell clusters via violin plot at the bottom of the page.

Supplemental figure 1

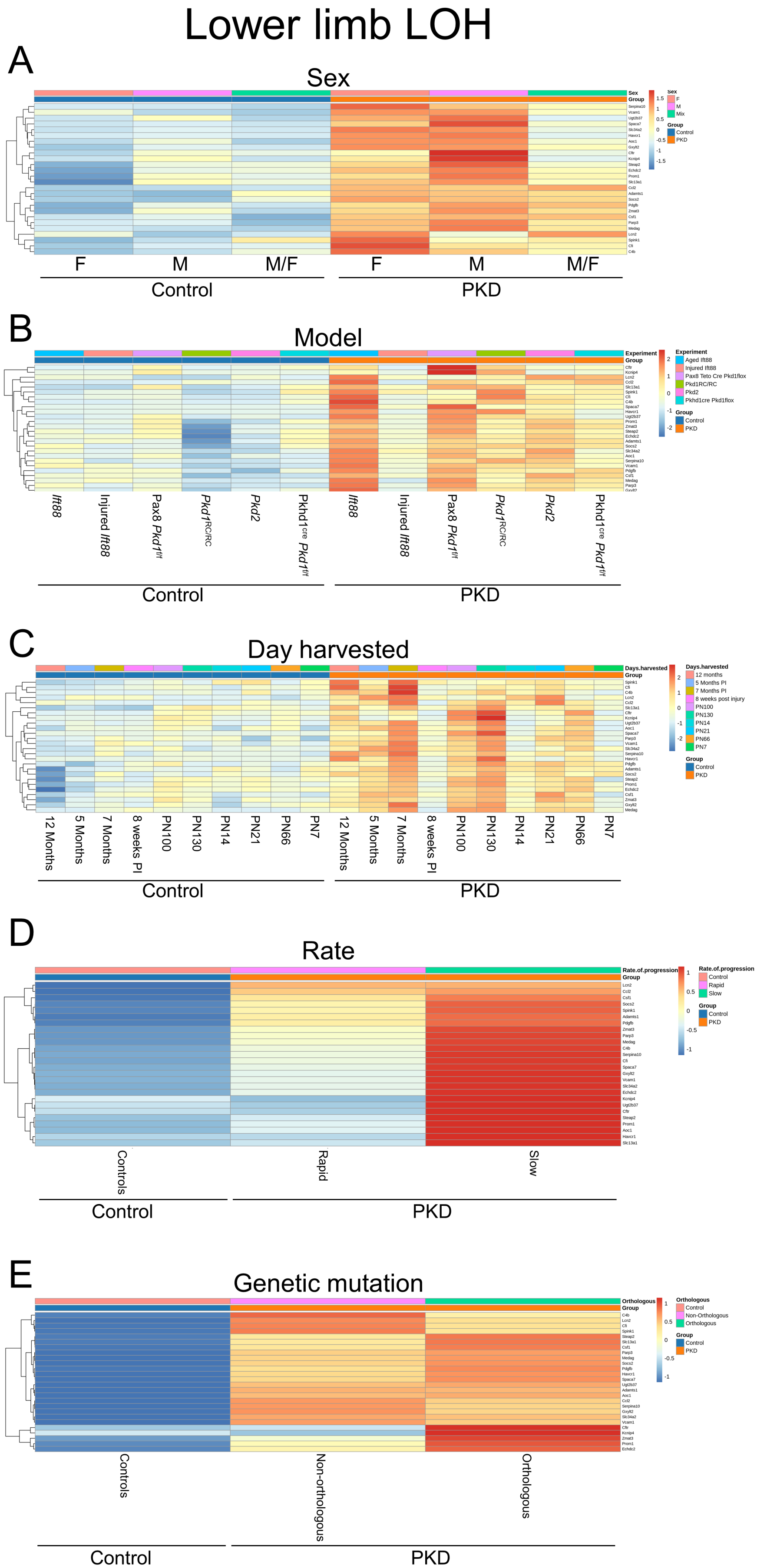

Supplemental figure 2

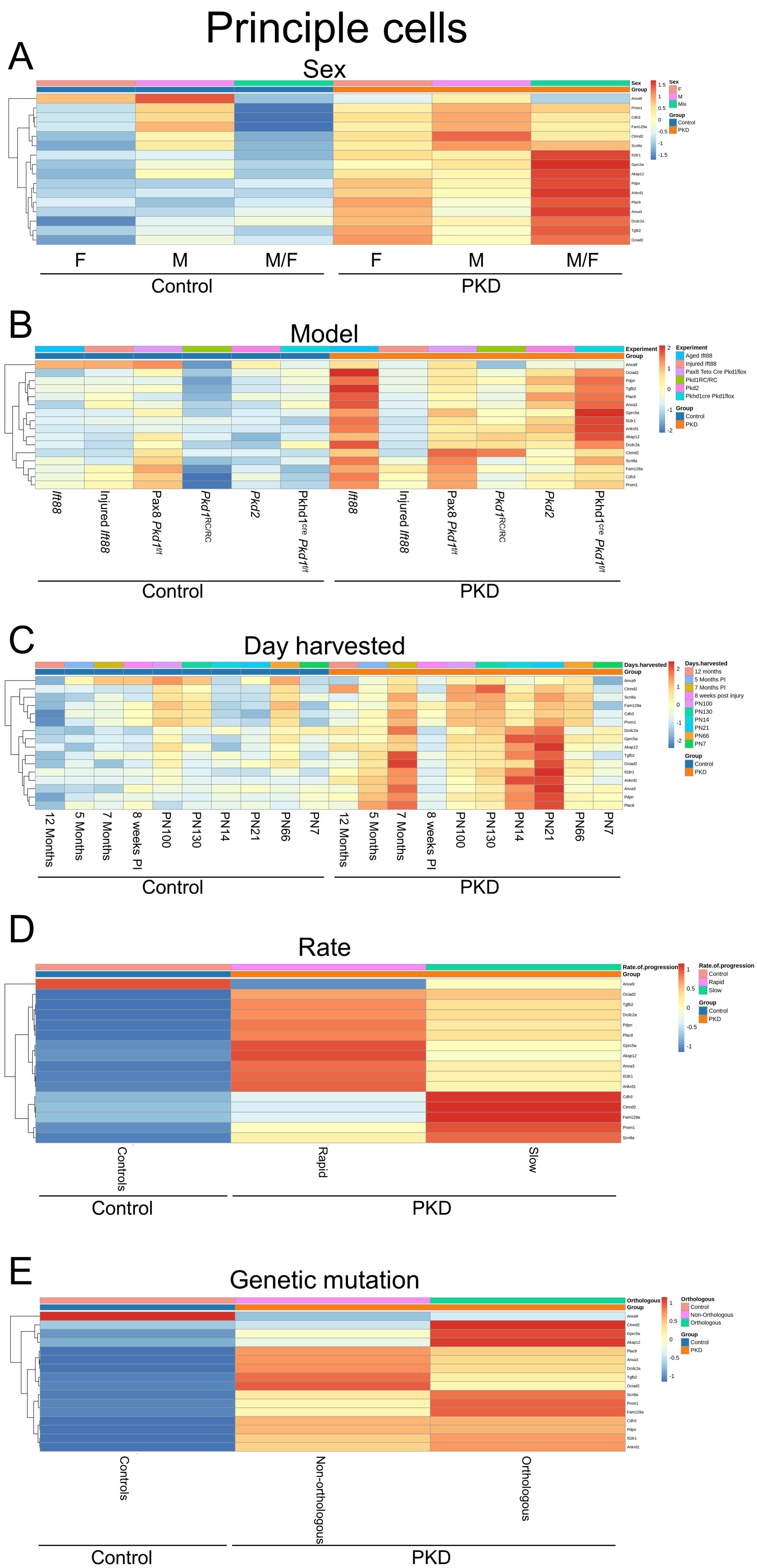

Supplemental figure 3

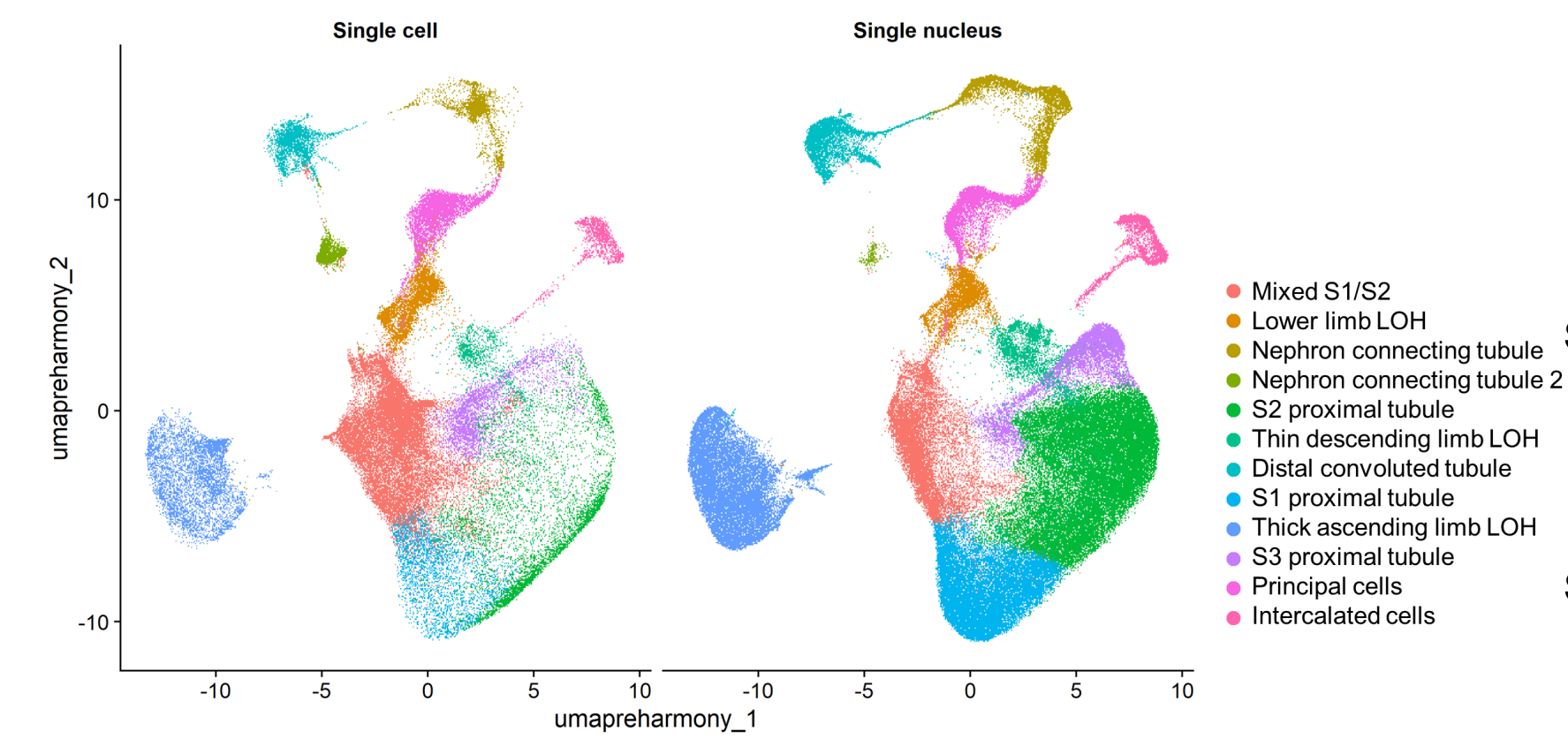

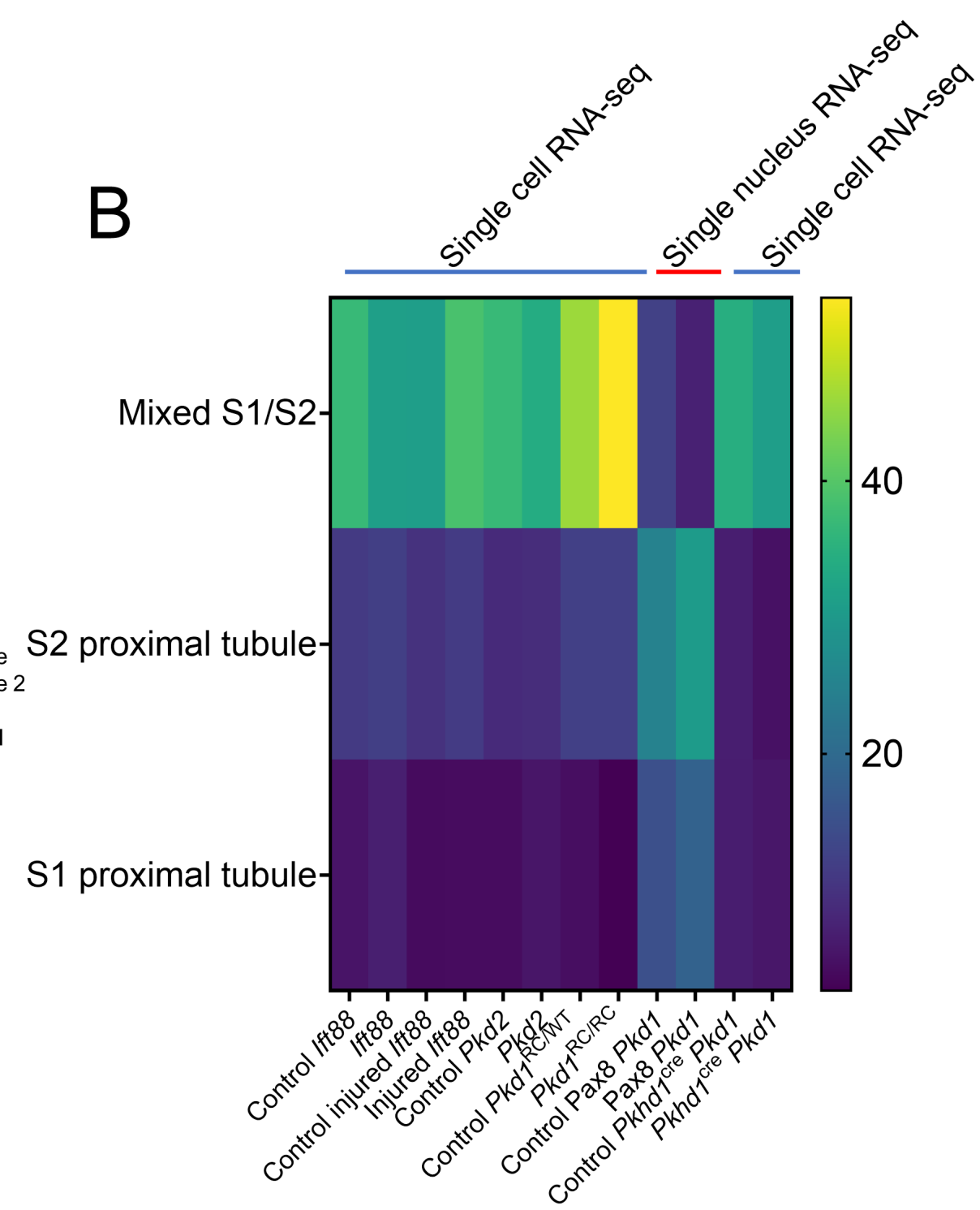

Supplemental figure 4

A

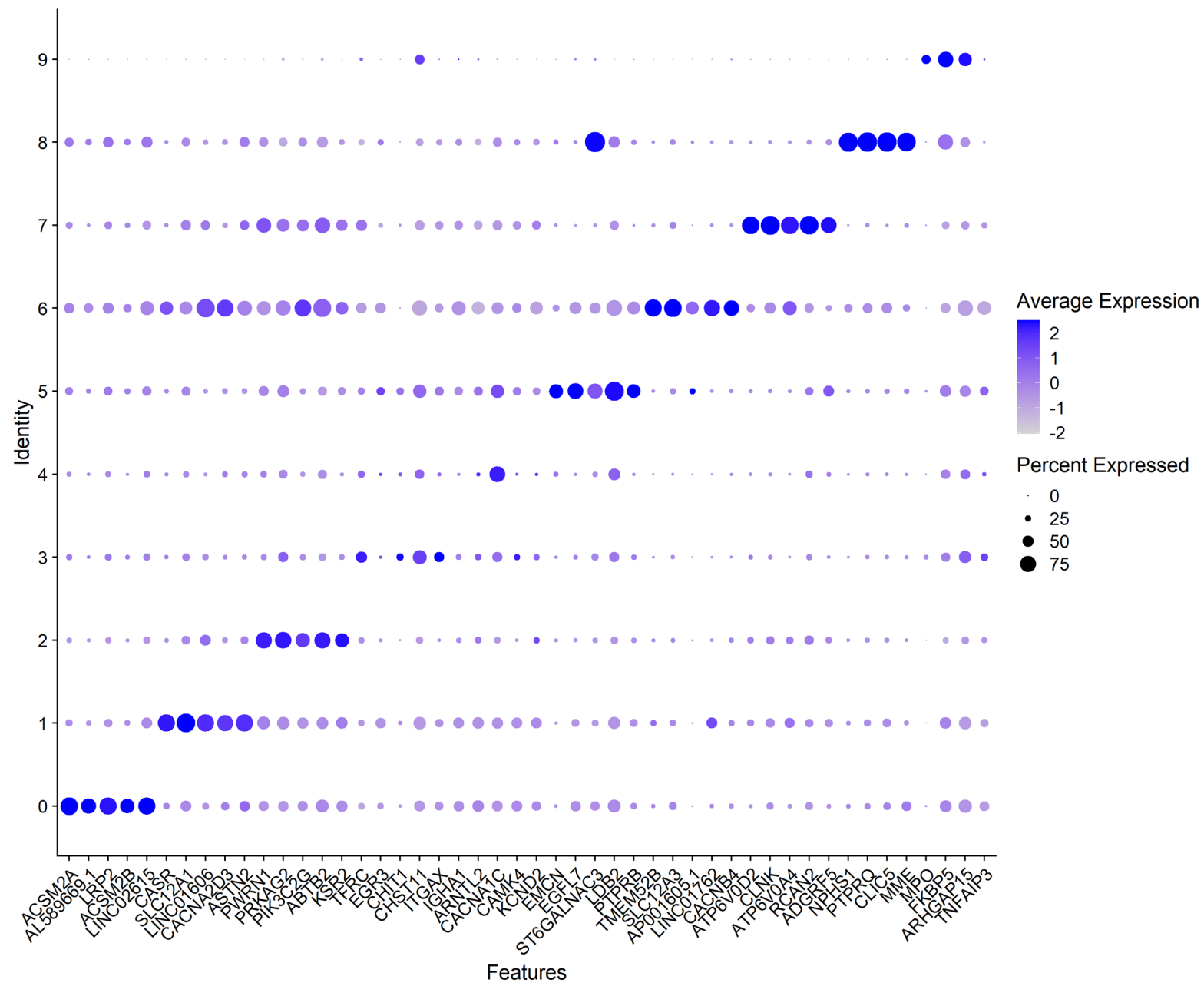

B

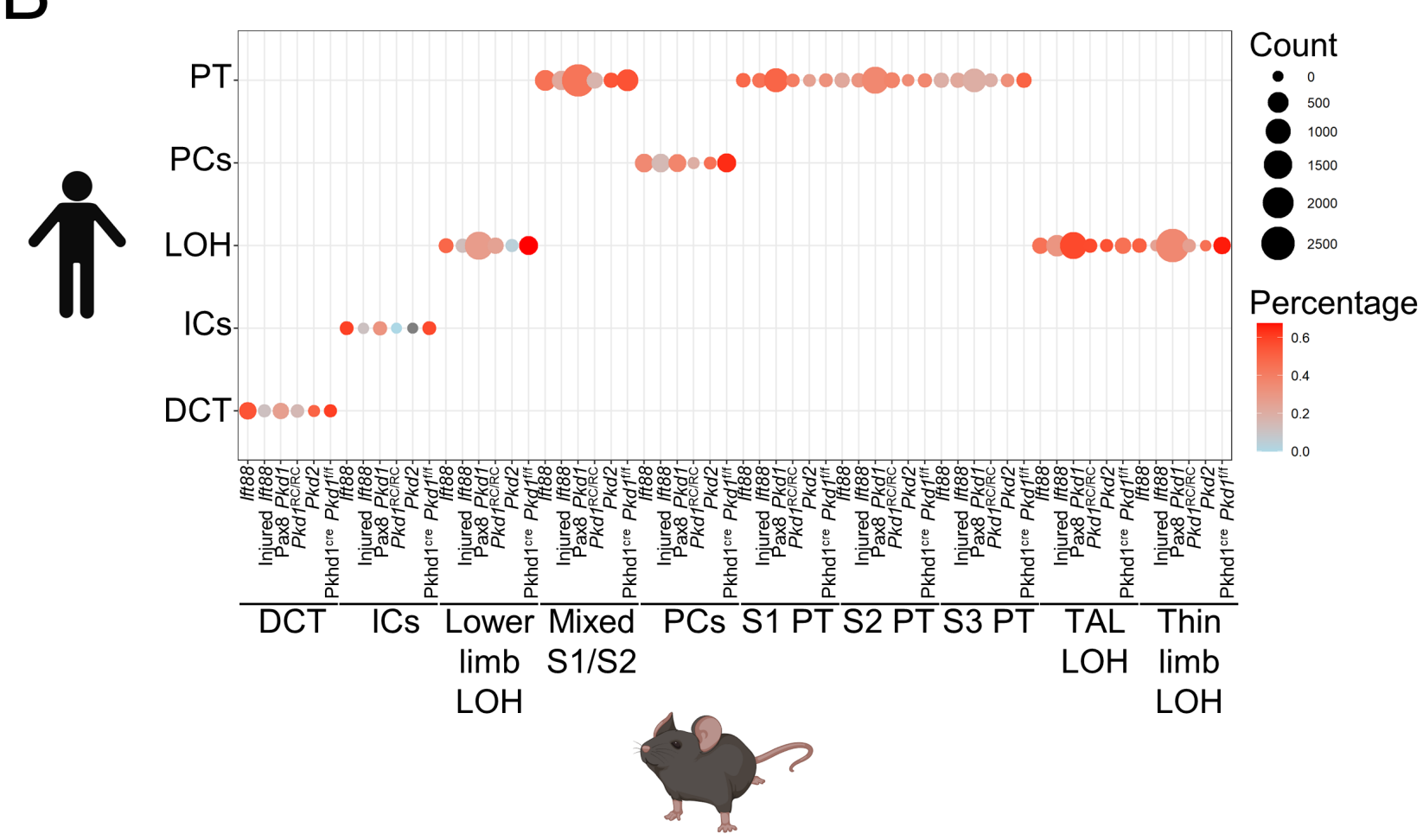

Supplemental Figure 5

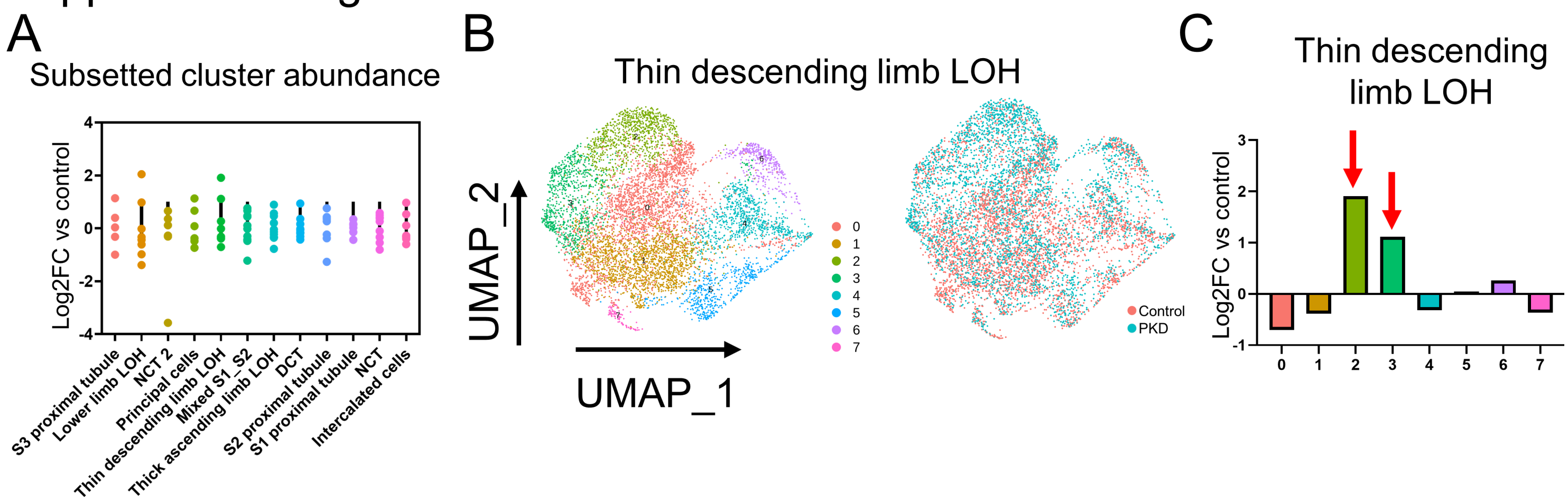

Supplemental figure 6

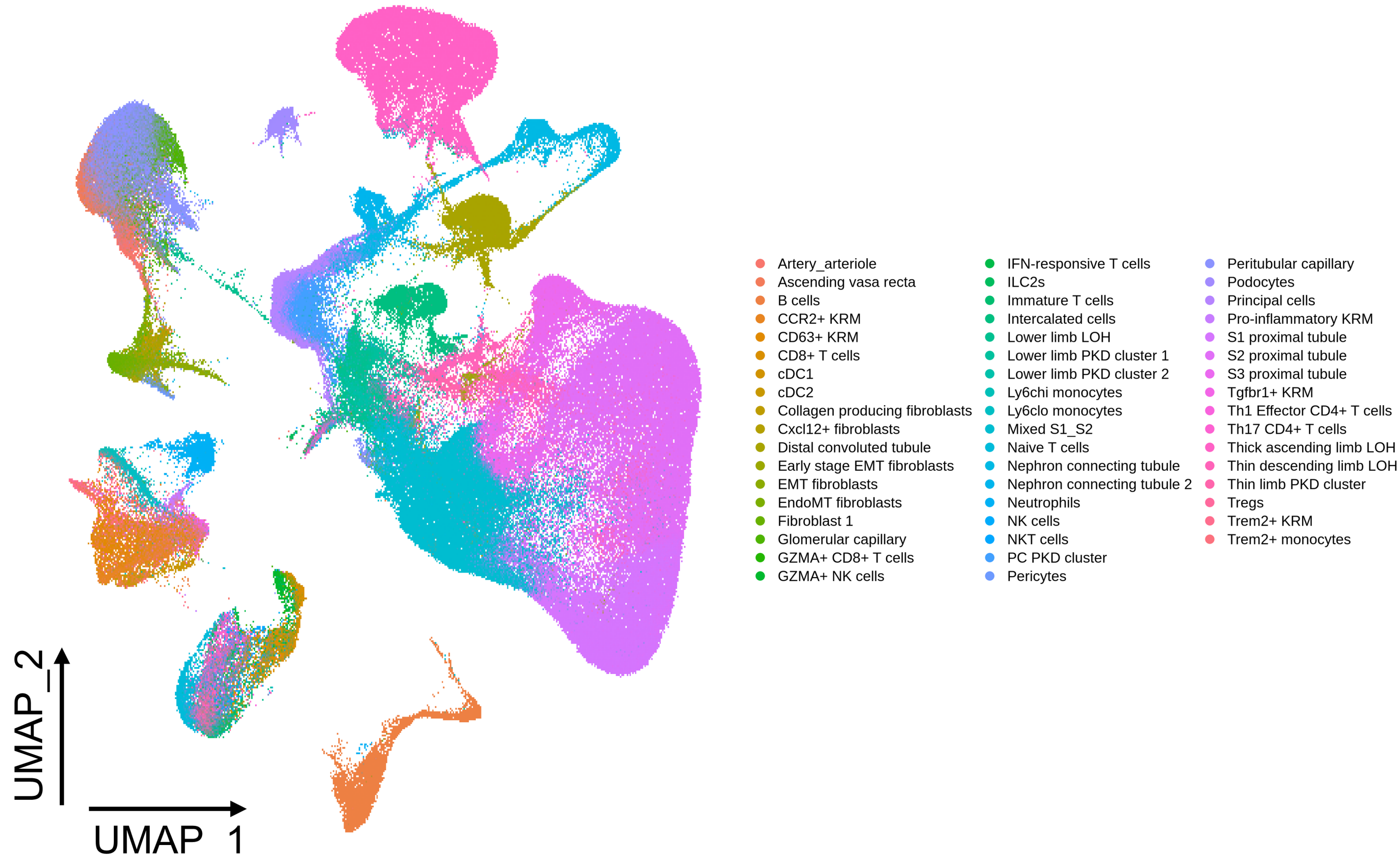

Supplemental figure 7

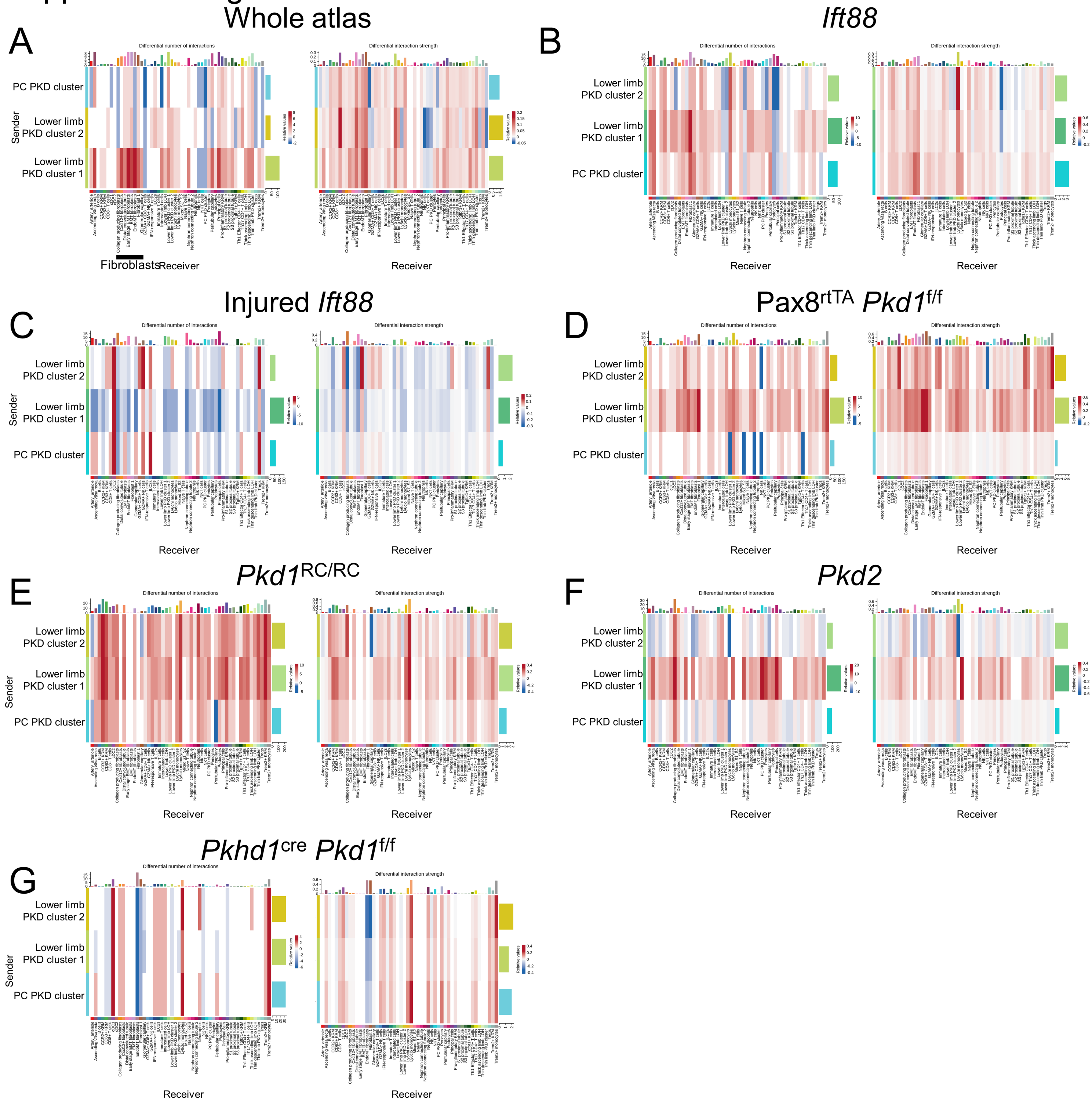

Supplemental figure 8

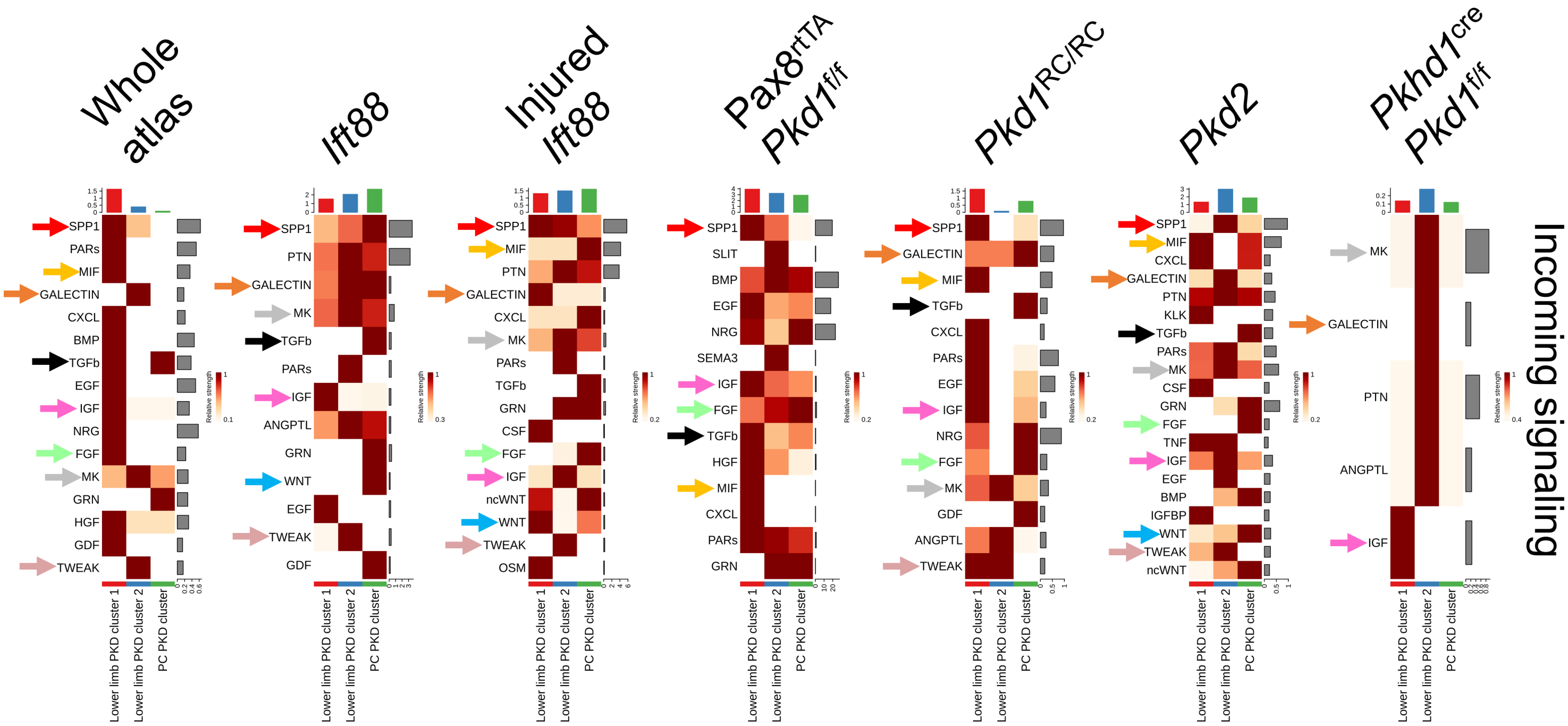

Supplemental figure 9

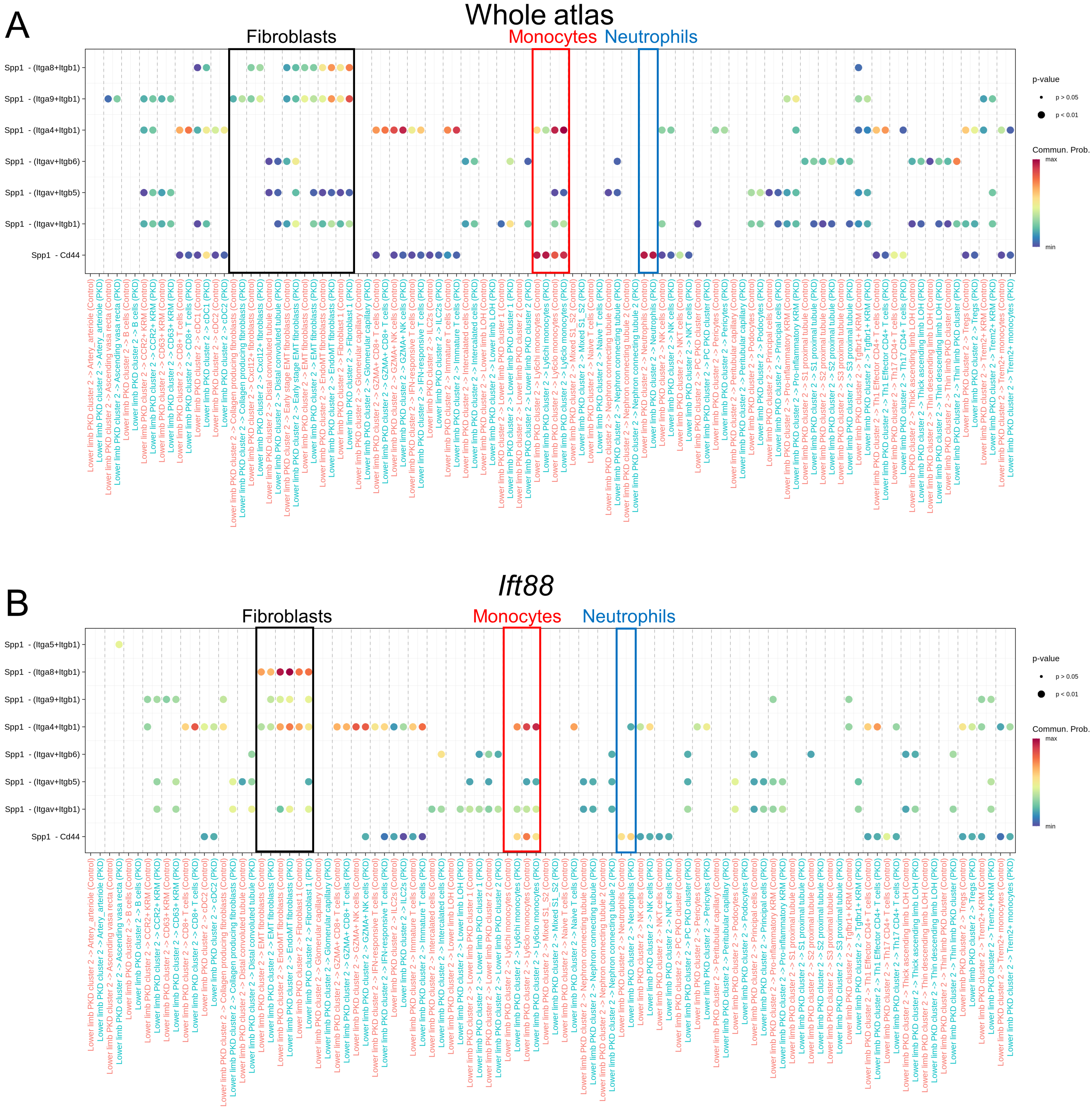

Supplemental figure 10

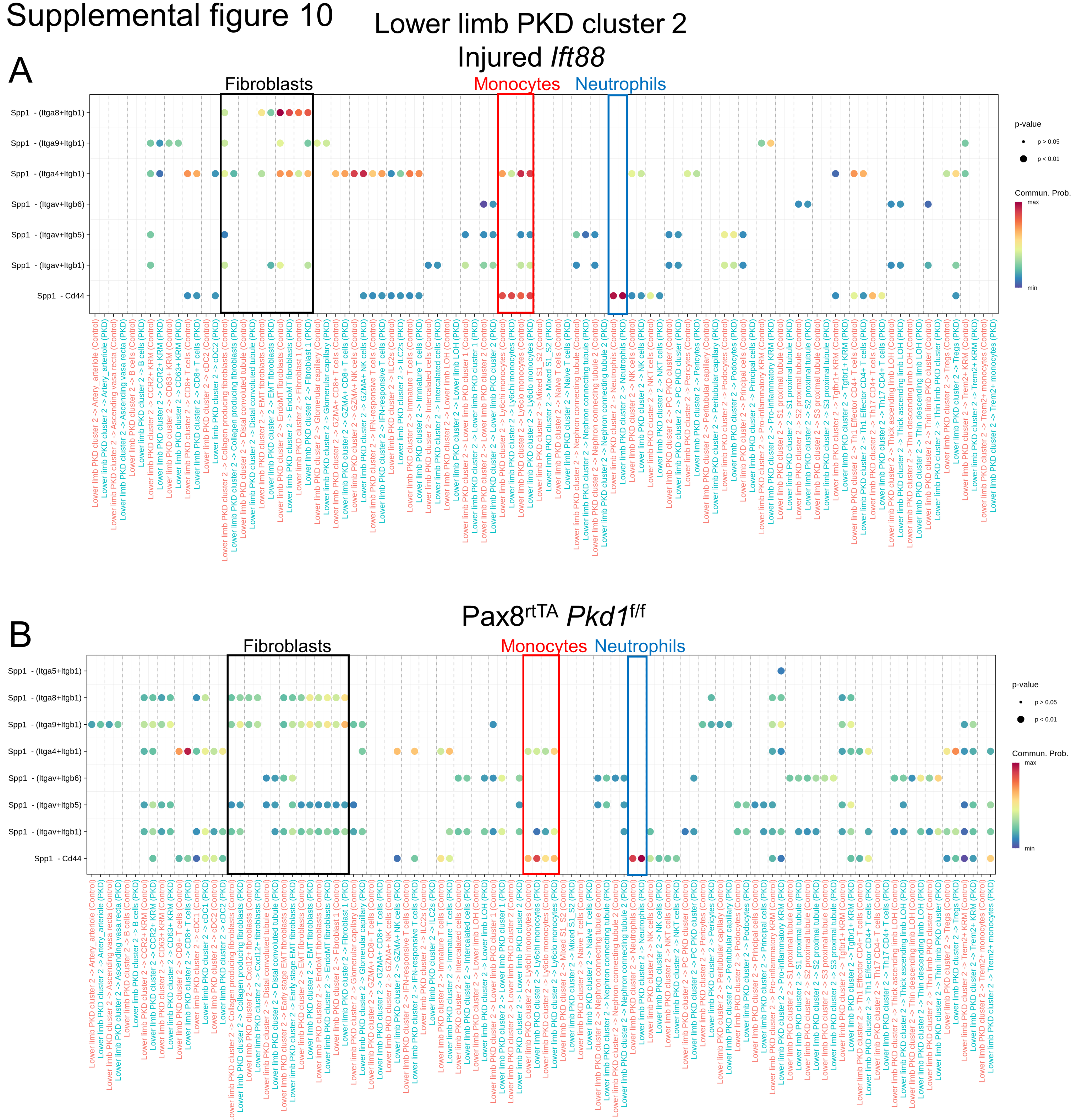

Supplemental figure 11

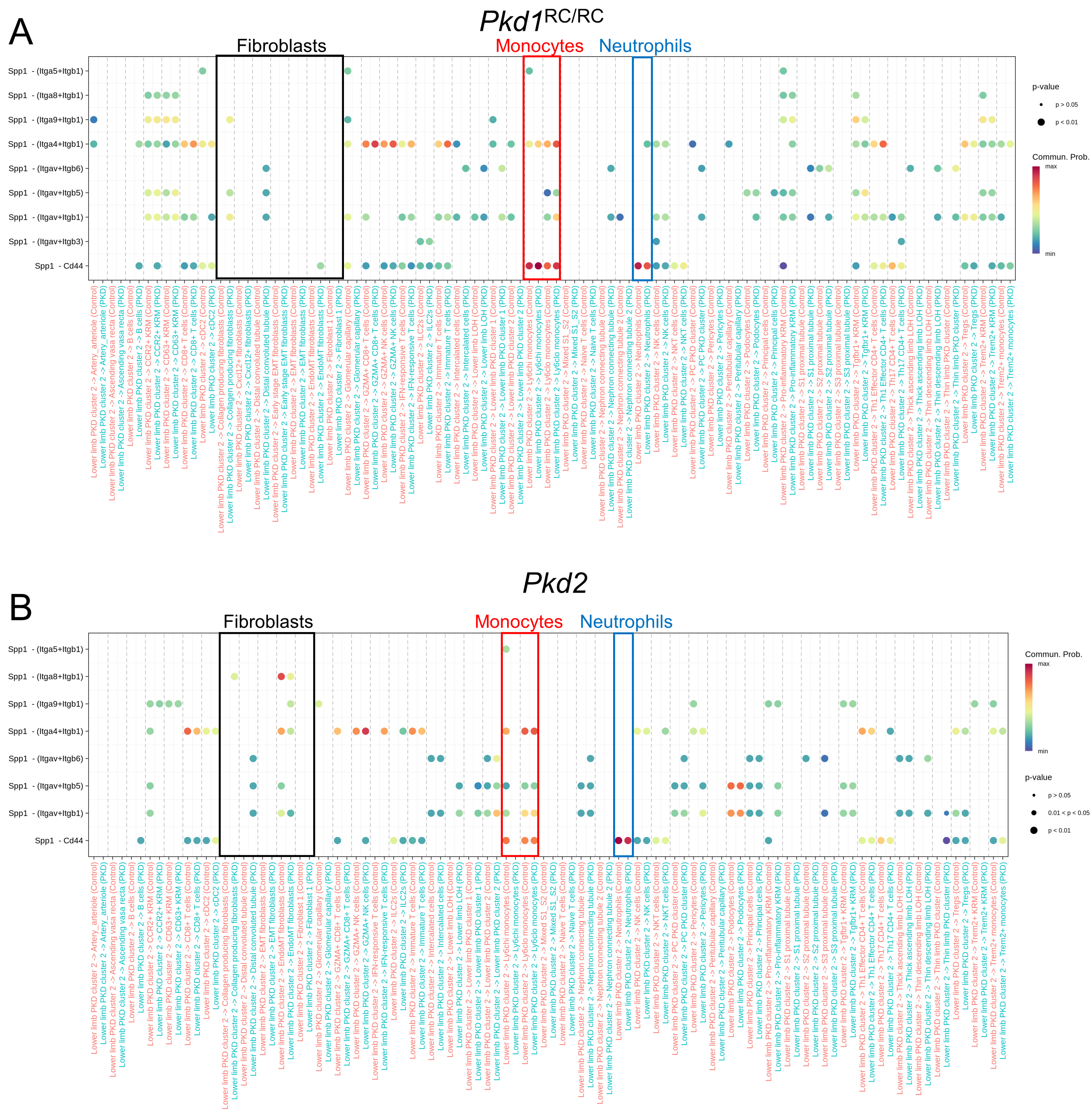

Supplemental figure 12

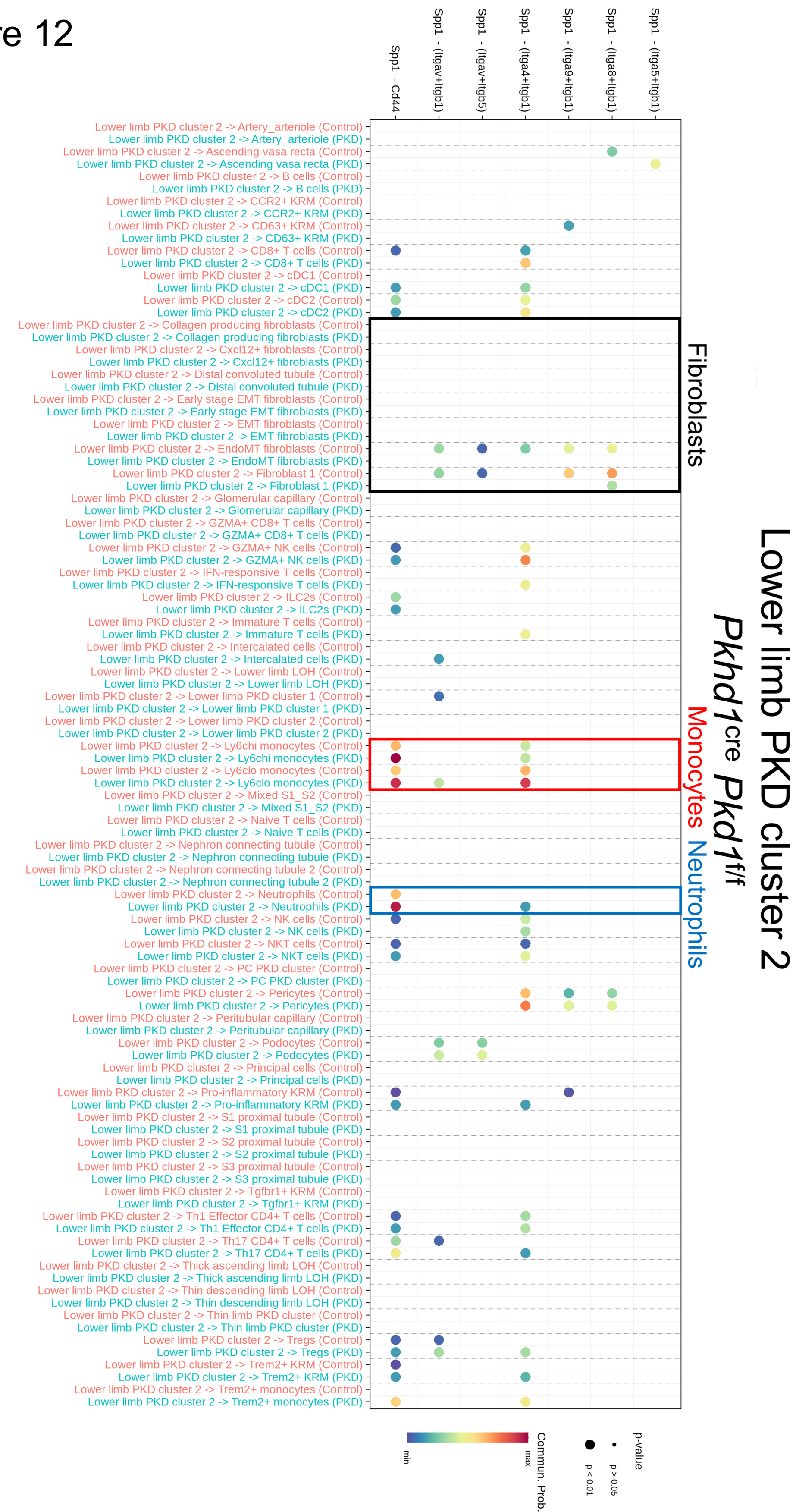

Supplemental figure 13

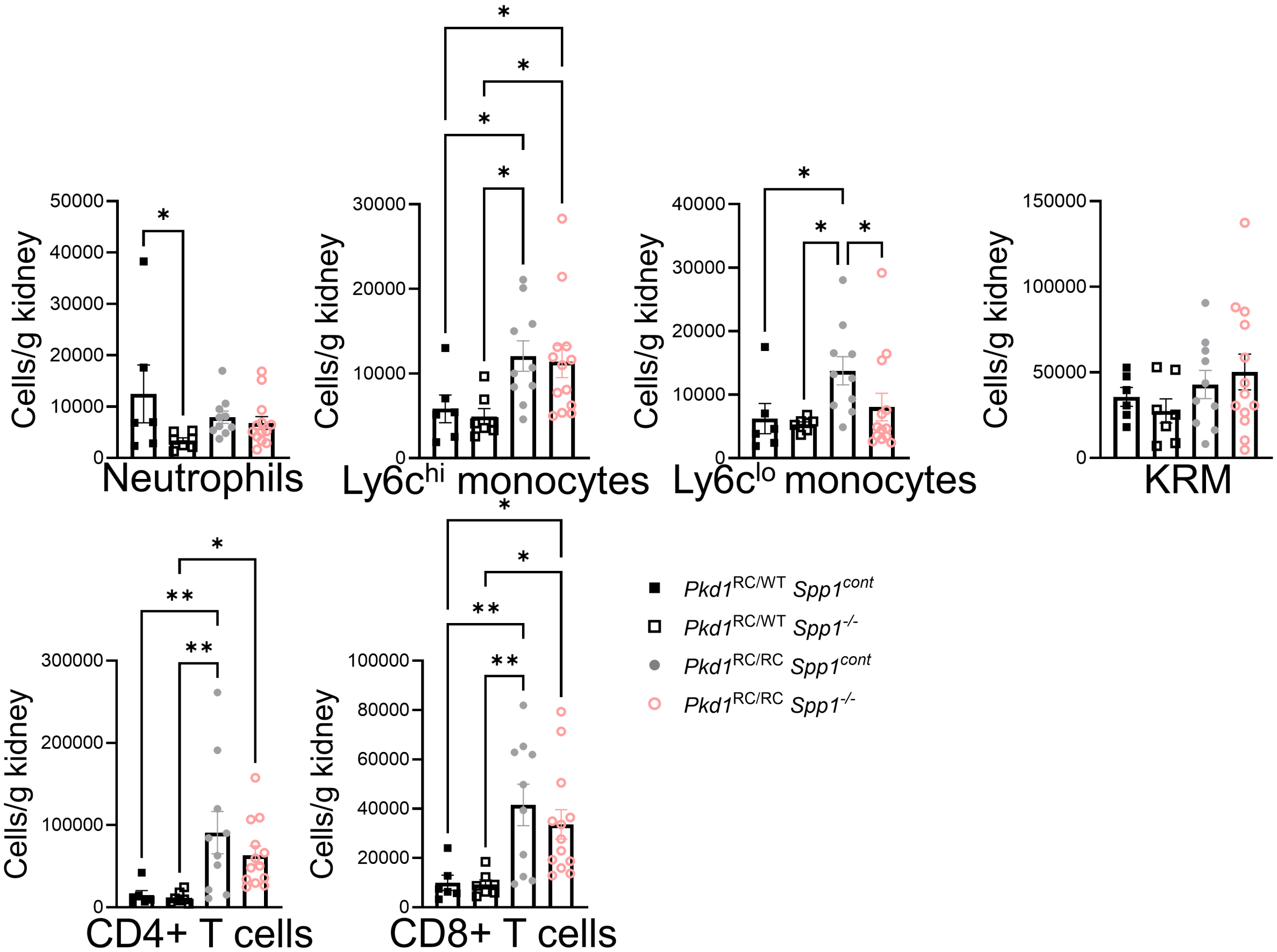

Supplemental figure 14

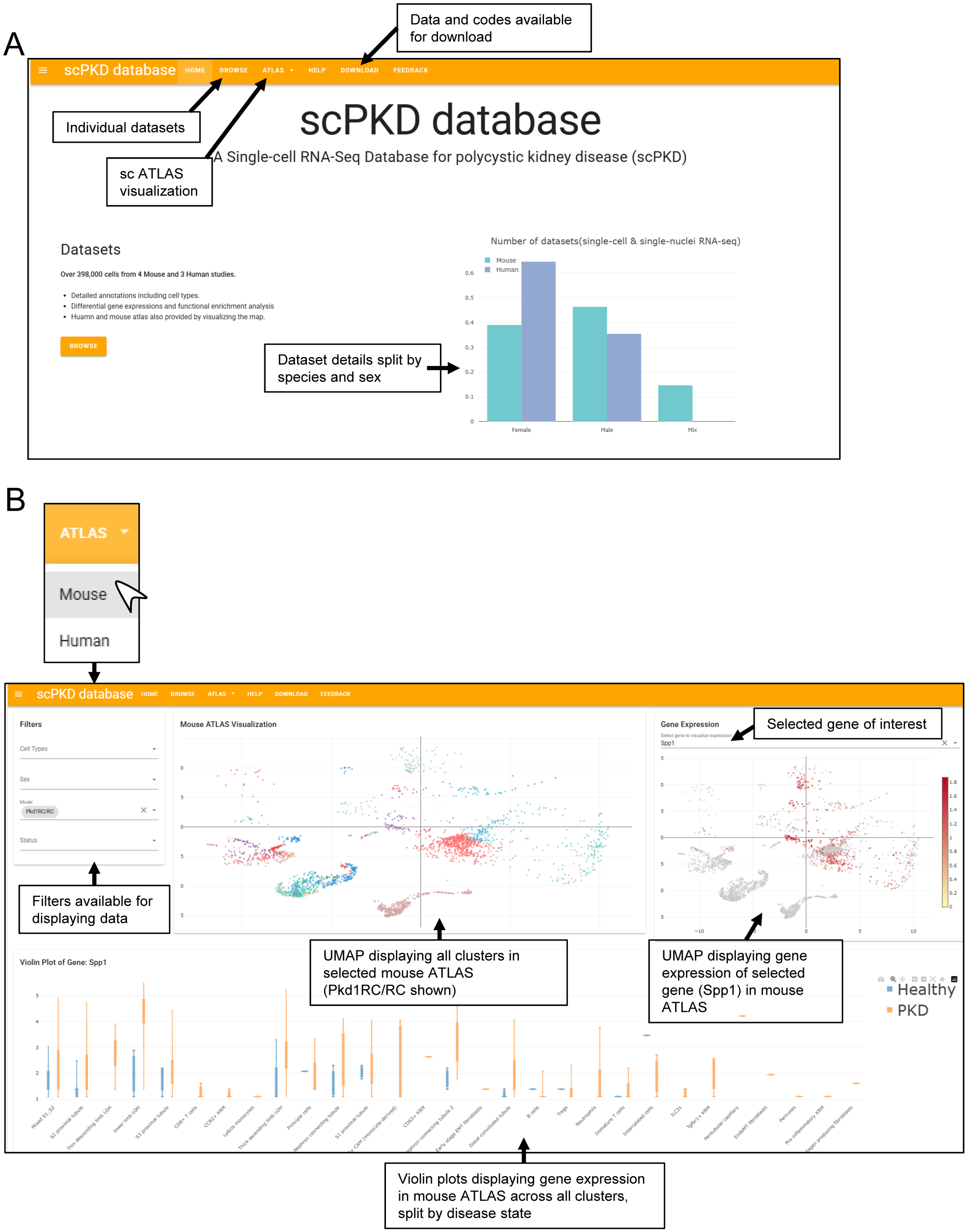

Supplemental Methods

Mouse models used for scRNAseq data:

<u>Ift88</u>: Seven to nine-week-old female C57BL/6J CAGGCre^{ERT2} Ift88$^{f/f}$ mice received a single intraperitoneal (IP) injection of tamoxifen at 6 mg/40g body weight for 3 consecutive days, as previously done by our lab(1, 2). CAGGCre^{ERT2} Ift88$^{f/f}$ mice were harvested ~6-7 months post tamoxifen induction and kidneys isolated for scRNAseq as described below. As scRNAseq is a discovery-based approach and not meant for rigid quantitative analysis, scRNAseq experiments were done on N=2 mice per group (2 control, 2 cystic; all female). Raw data are available in GEO (GSE193528) (1).

<u>Pkd2</u>: Seven to nine-week-old male and female C57BL/6J CAGGCre^{ERT2} Pkd2$^{f/f}$ mice received a single intraperitoneal (IP) injection of tamoxifen at 6 mg/40g body weight for 3 consecutive days. CAGGCre^{ERT2} Pkd2$^{f/f}$ mice were harvested ~4-5 months post tamoxifen induction and kidneys isolated for scRNAseq as described below. scRNAseq experiments were done on N=2 mice per group (2 control, 2 cystic; all female). Raw data are available in GEO (GSE297737).

Injured Ift88: Seven to nine-week-old male and female C57BL/6J CAGGCre^{ERT2} Ift88$^{f/f}$ mice received a single intraperitoneal (IP) injection of tamoxifen at 6 mg/40g body weight for 3 consecutive days. Three weeks post tamoxifen injection, mice were subjected to 30 minutes of sham surgery or unilateral ischemia reperfusion injury as described in our previous manuscript that generated the scRNAseq data(1). scRNAseq experiments were done on N=2 mice per group (2 control injured, 2 cystic sham operated, 2 cystic injured; all female). Raw data are available in GEO (GSE193528) (1).

<u>Pax8 teto^{cre} Pkd1$^{f/f}$</u>: Pax8 mice received doxycycline from P27 to P56 followed by harvest of kidneys at P66, P100, P130 as described(3). scRNAseq experiments were done on N=2-3 mice per group (P66: 2 control, 2 cystic; P100: 3 control, 3 cystic; P130: 3 control, 3 cystic; all male). Raw scRNAseq data was obtained from GEO (GSE268494) (3).

<u>Pkhd1^{cre} Pkd1$^{f/f}$</u>: Kidneys were harvested at P7, P14, P21 as previously described(4). scRNAseq experiments were done on 2-3 mice per group (male and female mix). Cellranger outputs were obtained from originating authors. No GEO data is available for these samples.

<u>Pkd1$^{RC/RC}$</u>: Control (Pkd1$^{RC/WT}$) and cystic (Pkd1$^{RC/RC}$) kidneys were harvested at one year of age and processed for scRNAseq as described below. scRNAseq experiments were done on N=2-3 mice per group (2 control, 3 cystic; 1M control, 1 F control; 1M cystic, 2F cystic). Raw data are available in GEO (GSE298379). Pkd1$^{RC/RC}$ mice were a kind gift from Dr. Peter Harris (Mayo Clinic, Rochester MN).

Sample prep for single cell RNA sequencing: For in house scRNAseq experiments (Pkd1$^{RC/RC}$, Pkd2), kidneys were minced and digested in 1ml of 2.5 mg/ml type II collagenase (Worthington, cat No; LS004176), 7.5 mg/ml B. licheniformis cold activated protease (Creative enzymes, Cat no: NATE-0633), and 125 U/ml DNase (Sigma-Aldrich, Cat no: D5025) in D-PBS for ~45 minutes at 12C as previously described(1). After digestion, kidney tissue was passed through individual 70-µm cell-strainers yielding single-cell suspensions. Cells were centrifuged at 300 X g for 5 minutes at 4ºC, resuspended in ACK red blood cell lysis buffer, and incubated at 37ºC for 5 minutes. After 5 minutes, 10 ml RPMI was added to each tube, cells were spun for 5 minutes at 4C, and cells were resuspended in 0.04% BSA with FC blocking solution (1:200, BioXcell;

catalog #: BE0307) for 30 minutes on ice. After blocking, cells were spun and incubated with the following antibodies in 0.04% BSA for 30 minutes: PE rat anti-mouse CD45 (catalog no. 12–0451, 30-F11; eBioscience), and Fixable Aqua Dead Cell Stain (catalog no. L34957; Invitrogen). Stained cells were then washed with 0.04% BSA and resuspended in 0.04% BSA for sorting. We sorted ~25,000 live, CD45+ or CD45- cells into individual BSA coated tubes. Cells were counted and approximately 5,000 cells from each group were combined into a single tube followed by performing bead emulsion and 10X genomics as described below.

10X genomics: 10x Chromium single cell libraries were prepared according to the standard protocol outlined in the manual. Briefly, sorted single cell suspension, 10x barcoded gel beads, and oil were loaded into Chromium™ Single Cell Chip G to capture single cells in nanoliter-scale oil droplets by Chromium™ Controller and to generate Gel Bead-In-EMulsions (GEMs). We used "Chromium™ Single Cell 3′ GEM, Library & Gel Bead Kit v3.1" and chip G for these experiments. Full length cDNA libraries were prepared by incubation of GEMs in a thermocycler machine. GEMs containing cDNAs were broken and all single cell cDNA libraries were pooled together, cleaned using DynaBeads MyOne™ Silane beads (Fisher PN 37002D), and pre-amplified by PCR to generate sufficient mass for sequencing library construction. Sequencing libraries were constructed by following the steps: cDNA fragmentation, end repair & A-tailing, size selection by SPRIselect beads (Beckman Coulter, PN B23318), adaptor ligation, sample index PCR amplification, and a repeat of SPRIselect beads size selection. The final constructed single cell libraries were sequenced by Illumina Nextseq2000 machine with total reads per cell targeted for a minimum of 25,000.

Single cell sequencing data processing: The 10X Genomics Cellranger software (version 7.1.0), 'mkfastq', was used to create the fastq files from the sequencer. Following fastq file generation, Cellranger 'count' was used to align the raw sequence reads to the reference genome using STAR. The 'count' software created 3 data files (barcodes.tsv, features.tsv, matrix.mtx) that were loaded into the R package Seurat version 5.2.0 (5), which allows for selection and filtration of cells based on QC metrics, data normalization and scaling, and detection of highly variable genes. Next, we used DoubletFinder(6) and Soupx (7) to remove doublets and ambient RNA from the data prior to data integration. We followed the Seurat vignette (https://satijalab.org/seurat/pbmc3k_tutorial.html) to create the Seurat data matrix object for each individual object followed by combining individual replicates together using the 'merge' function. To generate the integrated scRNAseq atlas shown in Figure 1B, we used SCT-RPCA on the layered Seurat object. After subsetting stromal cells, we used SCT-Harmony and the previous RPCA-integration. After all integration and merging steps, we removed low quality cells by keeping all genes expressed in greater than 3 cells and cells with at least 200 detected copies. Cells with mitochondrial gene percentages over 50% and unique gene counts greater than 3,000 or less than 200 were discarded, as previously established in the kidney (1). The data was normalized using Seurat's 'NormalizeData' function, which uses a global-scaling normalization method, LogNormalize, to normalize the gene expression measurements for each cell to the total gene expression. Highly variable genes were then identified using the function 'FindVariableGenes' in Seurat. We also regressed out the variation arising from library size and percentage of mitochondrial genes using the function 'ScaleData' in Seurat. We performed principal component analysis (PCA) of the variable genes as input and determined significant PCs based on the 'JackStraw' function in Seurat. The first 10 PCs were selected as input for

Uniform Manifold Approximation and Projection (UMAP) dimensionality reduction using the functions 'FindClusters' and 'DimPlot' in Seurat. To identify differentially expressed genes in each cell cluster, we used the function 'FindAllMarkers' in Seurat on the normalized gene expression data. Detailed code for generating and analyzing scRNAseq data can be found in github as outlined in the "code availability" section below.

DecoupleR: DecoupleR (8) for pathway and transcription factor inference was done using the integrated Seurat object and the standard vignette available on github (https://saezlab.github.io/decoupleR/articles/pw_sc.html).

Differential expression testing using pseudobulked data: For the differential expression testing, raw counts for each cluster for each experimental condition were pseudobulked using the 'AggregrateExpression' function and the counts slot. Differential gene expression testing was done using DESeq2 and plotted using pheatmap. Due to the high variance across groups, we used both adjusted p value (<0.05) and non-adjusted p value (<0.05) when plotting differentially expressed genes.

CellChat analysis for cell-cell communication in scRNAseq data: CellChat (9) was performed on the fully annotated scRNAseq atlas as shown in Supplemental figure 9B using the standard vignette for analyzing multiple scRNAseq samples (https://htmlpreview.github.io/?https://github.com/jinworks/CellChat/blob/master/tutorial/Comparison_analysis_of_multiple_datasets.html). Briefly, we created separate CellChat objects for

control and PKD samples using the group metadata column followed by merging the two CellChat objects into a single object using the 'mergeCellChat' function. We then calculated the netcentrality scores using the 'netAnalysis_computeCentrality' function followed by performing downstream analysis using the standard vignette.

$Pkd1^{RC/RC}$ Spp1 knockout mice: $Pkd1^{RC/RC}$ $Spp1^{cont}$ or $Pkd1^{RC/RC}$ Spp1-/- mice were harvested at ~1 year of age. Sample size was calculated based on initial data assuming a 25% difference in cystic index, 20% deviation, $\alpha = 0.05$, and $\beta = 0.2$. The power calculation indicated a minimum of N=10 mice per group. Outliers were identified as having a z score greater than 3 using the outlier test. For the manuscript, one mouse in the $Pkd1^{RC/RC}$ $Spp1^{cont}$ and one mouse in the $Pkd1^{RC/RC}$ Spp1-/- group was excluded as it had a z score greater than 3.

Tissue processing. $Pkd1^{RC/RC}$ Spp1 mice were perfused with PBS to remove contaminating immune cells in the vasculature. The left kidney was removed and put into RPMI 1640 on ice while the right kidney was cut in half and specimens were immediately immersed in in 10%(wt/vol) NBF overnight at 4ºC. The next day, samples were either switched to 30% (wt/vol) sucrose overnight at 4 ºC for cryopreservation or 70% ethanol for paraffin embedding. Paraffin embedded tissue was sectioned into 5 μm slices for use in downstream applications.

Quantification of cystic severity: 5-μm sections were cut from paraffin-embedded kidneys and stained with hematoxylin-eosin (H&E). To identify cysts, we began by quantifying the average diameter and standard deviation of a normal tubule using control, Cre negative mice. Cysts were

identified as any opening (white space) that was greater than 3 standard deviations above the average diameter of a normal tubule. To ensure that we did not include blood vessels and other artifacts that were the result of tissue processing in our cystic calculations, we went back and individually analyzed all particles that were identified as cysts using ImageJ. After removal of non-cystic structures, we calculated cystic index as the total cystic area divided by the total area of the sectioned kidney. This quantification was done using at least ½ of the kidney for all animals. A technician blinded to the treatment modality performed the quantification and analysis.

Flow cytometry: Flow cytometry experiments were performed following published protocols(10). Briefly, animals were perfused with PBS before the left kidney was extracted and minced followed by enzymatic digestion for 30 minutes at 37 C. Digestion buffer consisted of 1mg/mL collagenase Type I (Sigma-Aldrich, Catalog#: C0130-100MG) and 100 U/ml DNase I (Sigma-Aldrich, Catalog#: D5025-15KU). Cells were filtered through a 70µm strainer (MidSci, Catalog#: 70ICS), red blood cells were lysed (ACK lysis buffer, Quality Biological, Catalog#: 10128-802) and cells were resuspended in 1% BSA in PBS containing Fc blocking solution. After 30 minutes on ice, $2x10^6$ cells were stained with the appropriate amount of primary antibody (Supplemental table 3). Following a 30-minute incubation at room temperature, cells are washed and fixed with 2% PFA on ice for 30 minutes. Lastly, cells are resuspended in PBS before being analyzed on a Cytek Aurora. All analysis were conducted using FlowJo V10.9.0 software.

Blood Urea Nitrogen (BUN) analysis. Blood was collected from mice prior to perfusion with PBS via cardiac puncture and placed in EDTA coated tubes (Monoject, Catalog number: 8881311743). Blood samples were allowed to rock at room temperature for ~30 minutes prior to processing. Collected blood was centrifuged for 10 minutes at 1200 × g and the supernatant was transferred to an Eppendorf tube and stored at −80°C. Resulting plasma was analyzed for changes in BUN using the QuantiChrom Urea Assay Kit (DIUR-100; BioAssay Systems) according to the manufacturer's instructions.

RNA scope in ADPKD tissue. The RNA Scope HiPlex v2 assay from ACD Biosystems was performed according to manufacturers instructions using FFPE tissue slices. Kidney sections were hybridized with one probes (Hs-SPP1-T2Cy3 550, cat#420101-T2) and counterstained with DAPI. Slides were imaged using a Nikon CSU-W1/SoRa spinning disk confocal microscope.

Code availability: All code for these experiments can be found in out labs github account (kzimmer1) under the repository name "Mouse-SingleCell-Atlas".

Blinding and randomization: All mice used for scRNAseq were randomized prior to treatments. On the day of harvest, the flow cytometry and scRNAseq was performed by a scientist who was blinded to experimental groups. Bead capture, cDNA library preparation, sequencing, file conversion, and FASTq file generation were performed by the flow cytometry core at UAB or OMRF in a blinded manner. Data were processed in R in a blinded manner until the cluster composition and cell numbers were revealed.

References

1. *Song C, et al. A Comprehensive Immune Cell Atlas of Cystic Kidney Disease Reveals the Involvement of Adaptive Immune Cells in Injury-Mediated Cyst Progression in Mice. J Am Soc Nephrol. 2022.*
2. *Zimmerman KA, et al. Tissue-Resident Macrophages Promote Renal Cystic Disease. J Am Soc Nephrol. 2019;30(10):1841-56.*
3. *Muto Y, et al. Multiomics profiling of mouse polycystic kidney disease progression at a single-cell resolution. Proc Natl Acad Sci U S A. 2024;121(43):e2410830121.*
4. *Li LX, et al. Single-Cell and CellChat Resolution Identifies Collecting Duct Cell Subsets and Their Communications with Adjacent Cells in PKD Kidneys. Cells. 2022;12(1).*
5. *Butler A, et al. Integrating single-cell transcriptomic data across different conditions, technologies, and species. Nat Biotechnol. 2018;36(5):411-20.*
6. *McGinnis CS, et al. DoubletFinder: Doublet Detection in Single-Cell RNA Sequencing Data Using Artificial Nearest Neighbors. Cell Syst. 2019;8(4):329-37 e4.*
7. *Young MD, and Behjati S. SoupX removes ambient RNA contamination from droplet-based single-cell RNA sequencing data. Gigascience. 2020;9(12).*
8. *Badia IMP, et al. decoupleR: ensemble of computational methods to infer biological activities from omics data. Bioinform Adv. 2022;2(1):vbac016.*
9. *Jin S, et al. Inference and analysis of cell-cell communication using CellChat. Nat Commun. 2021;12(1):1088.*
10. *Miller SJ, et al. Isolation and Flow Cytometry Analysis of Macrophages from the Kidney. Methods Mol Biol. 2024;2713:171-81.*
